# PKCδ-Mediated Nox2 Activation Promotes Fluid-Phase Pinocytosis of Antigens by Immature Dendritic Cells

**DOI:** 10.3389/fimmu.2018.00537

**Published:** 2018-03-26

**Authors:** Bhupesh Singla, Pushpankur Ghoshal, Huiping Lin, Qingqing Wei, Zheng Dong, Gábor Csányi

**Affiliations:** ^1^Vascular Biology Center, Medical College of Georgia, Augusta University, Augusta, GA, United States; ^2^Department of Cellular Biology and Anatomy, Medical College of Georgia, Augusta University, Augusta, GA, United States; ^3^Department of Pharmacology and Toxicology, Medical College of Georgia, Augusta University, Augusta, GA, United States

**Keywords:** dendritic cells, macropinocytosis, protein kinase C, NADPH oxidase, reactive oxygen species

## Abstract

**Aims:**

Macropinocytosis is a major endocytic pathway by which dendritic cells (DCs) internalize antigens in the periphery. Despite the importance of DCs in the initiation and control of adaptive immune responses, the signaling mechanisms mediating DC macropinocytosis of antigens remain largely unknown. The goal of the present study was to investigate whether protein kinase C (PKC) is involved in stimulation of DC macropinocytosis and, if so, to identify the specific PKC isoform(s) and downstream signaling mechanisms involved.

**Methods:**

Various cellular, molecular and immunological techniques, pharmacological approaches and genetic knockout mice were utilized to investigate the signaling mechanisms mediating DC macropinocytosis.

**Results:**

Confocal laser scanning microscopy confirmed that DCs internalize fluorescent antigens (ovalbumin) using macropinocytosis. Pharmacological blockade of classical and novel PKC isoforms using calphostin C abolished both phorbol ester- and hepatocyte growth factor-induced antigen macropinocytosis in DCs. The qRT-PCR experiments identified PKCδ as the dominant PKC isoform in DCs. Genetic studies demonstrated the functional role of PKCδ in DC macropinocytosis of antigens, their subsequent maturation, and secretion of various T-cell stimulatory cytokines, including IL-1α, TNF-α and IFN-β. Additional mechanistic studies identified NADPH oxidase 2 (Nox2) and intracellular superoxide anion as important players in DC macropinocytosis of antigens downstream of PKCδ activation.

**Conclusion:**

The findings of the present study demonstrate a novel mechanism by which PKCδ activation *via* stimulation of Nox2 activity and downstream redox signaling promotes DC macropinocytosis of antigens. PKCδ/Nox2-mediated antigen macropinocytosis stimulates maturation of DCs and secretion of T-cell stimulatory cytokines. These findings may contribute to a better understanding of the regulatory mechanisms in DC macropinocytosis and downstream regulation of T-cell-mediated responses.

## Introduction

Dendritic cells (DCs) are professional antigen-presenting cells (APCs) that initiate and direct adaptive immune responses (1–3). Immature DCs (iDCs) in peripheral tissue constantly sample their surroundings for antigens. Following antigen internalization, iDCs exhibit dramatic functional and morphological changes, called *maturation*, which optimizes antigen processing and maximizes antigen presentation to naïve T cells. During maturation, iDCs acquire a phenotype of professional APCs, synthetize and secrete T-cell costimulatory cytokines, increase plasma membrane expression of CD86 and CD80, and translocate major histocompatibility complex class I and II (MHC I/II) molecules from the late endocytic compartments to the plasma membrane (4). Mature DCs migrate to draining lymph nodes and present the processed antigenic peptides on MHC I/II to T lymphocytes to initiate antigen-specific immune responses (2). The ability of mature DCs to internalize and process antigens is inhibited to restrict their ability to present additional antigens encountered after the initial immunogenic stimulus (5).

Immature DCs capture antigens by several distinct mechanisms, including “non-specific” internalization by macropinocytosis and “specific” uptake *via* receptor-mediated endocytosis and phagocytosis (4). Importantly, the endocytic process by which antigens are internalized not only determines the intracellular trafficking of the antigen but also influences the type of T-cell epitope being presented on MHC molecules (6). Previous studies showed that macropinocytosis is distinct in many ways from receptor-mediated endocytosis and phagocytosis (7). Indeed, phagocytosis and receptor-mediated endocytosis are strictly ligand-receptor-driven processes (4, 8), while macropinocytosis is characterized by receptor-independent internalization of extracellular fluid and pericellular solutes (4, 9). Phagocytosis is initiated by recognition and binding of the particle to the plasma membrane, followed by localized actin remodeling, formation of a phagocytic cup around the particle and its subsequent internalization into the phagosome (7). Unlike phagocytosis, receptor-mediated endocytosis is largely an actin-independent process in mammalian cells (10). In receptor-mediated endocytosis, specific cell surface receptors, such as C-type lectin receptors, Fcγ and Fcε receptors mediate antigen internalization by DCs (11). On the contrary, macropinocytosis involves particle-independent, global activation of the actin cytoskeleton resulting in extensive plasma membrane ruffling over the entire surface of the cell. Some of the membrane ruffles curve into O-shaped macropinocytotic cups and close or fuse with the non-extended plasma membrane, leading to macropinosome formation and non-specific internalization of extracellular fluid and associated solutes (4, 7, 9).

Previous studies demonstrated that membrane ruffling and macropinocytosis can be stimulated by various growth factors, including epidermal growth factor (12) and hepatocyte growth factor (HGF) (13), cytokines (14, 15), and phorbol esters (15, 16). Although the precise signaling mechanisms responsible for stimulation of macropinocytosis in DCs and other cell types are incompletely defined, phosphatidylinositol phosphates have been shown to play an important role (17). Plasma membrane phosphatidylinositol 4,5-bisphosphate [PI(4,5)P_2_] regulates the activity of a number of actin-binding proteins and, thus, plays an important role in controlling submembranous actin polymerization and reorganization during macropinocytosis (10). PI(4,5)P_2_ is phosphorylated to PI(3,4,5)P_3_ by phosphatidylinositol-3-kinase (PI3K) followed by recruitment and activation of small GTPase Rac1 and Rab5 to mediate cup closure and initiate macropinosome formation (10, 18). Furthermore, PI(4,5)P_2_ is a substrate for phospholipase C, which produces two important signaling molecules: diacylglycerol (DAG) and inositol trisphosphate (IP_3_) (17). Recent studies by our lab and others have demonstrated that DAG-mediated protein kinase C (PKC) activation in macrophages plays an important role in macropinocytosis (15, 17). The PKC family has been categorized into three groups, namely the DAG/Ca^2+^-dependent classical (α, β, and γ), DAG-dependent novel (δ, ε, η, and θ), and DAG/Ca^2+^-independent atypical (λ, ι, μ, and ζ) PKC isoforms. Importantly, the PKC isoforms differ in their mechanism of activation, substrates, and signaling in the cell (19–21). The specific PKC isoform(s) mediating DC macropinocytosis of antigens and the signaling mechanisms downstream of PKC leading to macropinocytosis are currently unknown.

The NADPH oxidases (Noxs) are transmembrane proteins that transfer electrons across biological membranes to reduce oxygen to superoxide anion $\left({\text{O}}_{2}^{{\;}^{•-}}\right)$ or its dismuted form, hydrogen peroxide (H_2_O_2_) (22). The Nox family consists of seven members, namely Nox1–Nox5, dual oxidase (DUOX) 1, and DUOX2. Nox2, the prototype isoform of the Nox family, consists of flavocytochrome b558, an integral membrane heterodimer comprising gp91*^phox^* and p22*^phox^*, and four cytoplasmic protein subunits: p47*^phox^*, p67*^phox^*, p40*^phox^*, and Rac1 (23). Nox2 is dormant in resting cells and becomes rapidly activated on stimulation by growth factors, cytokines, and phorbol esters (15, 24–26). During activation, PKC mediates phosphorylation of p47*^phox^*, leading to its translocation to the catalytic core of Nox2 along with the cytosolic subunits, followed by NADPH-mediated electron transfer and ${\text{O}}_{2}^{{\;}^{•-}}$ generation (26). Although signal transduction mediators upstream of Nox2 activation, such as PKC and Rac1, play an important role in macropinocytosis (10) and Nox2 activators also stimulate macropinocytosis (13, 15, 26, 27), the role of Nox2 in DC macropinocytosis has not been previously investigated.

The goal of the present study was to identify the specific PKC isoform(s) involved in DC macropinocytosis. We also tested the hypothesis that Nox2 activation downstream of PKC contributes to DC macropinocytosis of antigens, leading to their maturation and secretion of T- cell stimulatory cytokines. The data shown herein demonstrate the involvement of a previously unidentified mechanism by which DAG-inducible PKCδ *via* stimulation of Nox2 activity promotes DC macropinocytosis of antigens. PKCδ–Nox2-mediated antigen macropinocytosis induced MHC II translocation to the membrane, increased expression of CD86, and secretion of various T-cell stimulatory cytokines. These findings may be relevant for various immune disorders involving elevated uptake of antigens by DC, and ultimately leading to enhanced T-cell-mediated inflammatory responses.

## Materials and Methods

### Reagents and Antibodies

Mouse recombinant granulocyte-macrophage colony-stimulating factor (GM-CSF), interleukin-4 (IL-4), recombinant murine FLT3-Ligand (FLT3L) and HGF were obtained from Peprotech (Rocky Hill, NJ, USA). FM™ 4-64 Dye and Alexa Fluor 488-conjugated ovalbumin (OVA) was procured from Molecular Probes (Eugene, OR, USA). Calphostin C, superoxide dismutase (SOD), diphenyleneiodonium chloride (DPI), LY294002, 5-(N-ethyl-N-isopropyl) amiloride (EIPA), phorbol 12-myristate 13-acetate (PMA), cytochalasin D, lipopolysaccharide (LPS), and OVA were purchased from Sigma-Aldrich (St. Louis, MO, USA). Protease and phosphatase inhibitor cocktail tablets were purchased from Roche Diagnostics GmbH (Mannheim, Germany). FITC-OVA and dihydroethidium (DHE) were obtained from Thermofisher Scientific (Grand Island, New York, NY, USA). EUK-134 was purchased from Cayman Chemical (Ann Arbor, MI, USA). L-012 was obtained from Wako Chemicals (Richmond, VA, USA). PE/Cy7-CD11c (clone N418), APC-CD11c (clone N418), FITC-MHCII (clone M5/114.15.2), APC-CD86 (clone GL1) monoclonal antibodies, and respective isotype controls were purchased from Thermofisher Scientific.

### Animals

All experimental protocols were approved by the Institutional Animal Care and Use Committee of Augusta University and conducted in accordance with the National Institutes of Health Guide for the Care and Use of Laboratory Animals. Eight- to ten-week-old male, C57BL/6 (wild type) and Nox2^y/−^ mice were purchased from The Jackson Laboratory (Bar Harbor, ME, USA). Breeding pairs of PKCδ^+/−^ mice (kindly provided by Dr. Zheng Dong, Augusta University, USA), were used to generate PKCδ^+/−^, PKCδ^−/−^, and PKCδ^+/+^ mice. Mice were housed at constant temperature (21–23°C) with *ad libitum* access to standard rodent chow and water, and were maintained in 12 h light-dark cycles. Mice were anesthetized (isoflurane inhalation, 3%) and sacrificed by cervical dislocation and exsanguination.

### Generation of Mouse Bone Marrow-Derived Immature Dendritic Cells (BMiDCs) and Isolation of Splenic DCs

#### Bone Marrow-Derived Immature Dendritic Cells

The femur and tibia of sacrificed mice were cleaned of adherent muscle and connective tissue. Bone marrow was flushed from bone marrow cavities using a 27-gauge needle syringe containing Harvest Buffer [phosphate-buffered saline (PBS) and 2% heat-inactivated FBS] (28). Cells were plated in RPMI-1640 medium containing 10% FBS and differentiated into DCs with GM-CSF (20 ng/mL) and IL-4 (2 ng/mL) for 7 days. After 7 days, non-adherent and loosely attached cells were collected by gentle washing and used for experiments. For FLT3L-iDCs, mouse bone marrow cells were differentiated in complete RPMI-1640 medium containing 200 ng/mL mouse recombinant FLT3L for 9 days without disturbing (29). iDCs were identified by CD11c positivity, low- to intermediate expression of MHC II, and low expression of CD86. LPS (500 ng/mL, 24 h), a known inducer of DC maturation, was used to validate the immature phenotype of differentiated DCs.

#### Splenic DCs

Isolated mouse spleens were minced and digested with collagenase/dispase solution (collagenase: 0.1 U/mL, dispase: 0.8 U/mL) (Roche Diagnostics Corporation, Indianapolis, IN, USA) for 45 min at 37°C. After digestion, the cell suspension was filtered through a 70-µm cell strainer and CD11c^+^ cells were isolated using mouse CD11c MicroBeads (Miltenyi Biotec Inc. Auburn, CA, USA) according to the manufacturer’s instructions.

### Flow Cytometry

Splenic DCs and BMiDCs were incubated with FITC-OVA (50 µg/mL; 45,000 MW) in the presence and absence of PMA (1 µM) for 1 and 5 h, respectively. In separate experiments, BMiDCs were incubated with vehicle or HGF (100 ng/mL) and FITC-OVA internalization was investigated. After the incubation time, cells were washed twice with ice-cold PBS, stained with PE/Cy7- or APC-labeled CD11c (or respective isotype controls) monoclonal antibodies, fixed in 2% paraformaldehyde (PFA), resuspended in fluorescence-activated cell sorting (FACS) buffer (2% BSA and 0.01% sodium azide in PBS) and analyzed using a Becton Dickinson FACSCalibur flow cytometer. CD11c^+^ cells were gated and analyzed for FITC-OVA uptake.

### Confocal Microscopy

Bone marrow-derived immature dendritic cells were plated on poly-d lysine-coated glass bottom dishes and allowed to adhere for 90 min. Cells were incubated with FM™ 4-64 (5 µg/mL, 10 min) to label the plasma membrane and treated with Alexa Fluor 488-OVA (50 µg/mL; 45,000 MW). Macropinocytosis was stimulated by incubation with PMA (1 µM, 30 min). Accumulation of Alexa Fluor 488-OVA in membrane-derived macropinosomes was visualized using a Zeiss 780 inverted confocal microscope in live cells.

In separate experiments, BMiDCs were treated with FITC-OVA, incubated with vehicle or PMA for 5 h, and centrifuged at 300 × *g* for 10 min to sediment on gelatin-coated coverslips. Cells were fixed in 2% PFA, permeabilized with 0.1% Triton X-100, and stained with TRITC-phalloidin (Sigma-Aldrich, St. Louis, MO, USA) and Hoechst 33342 (Life Technologies, Grand Island, NY). Images were captured using a Zeiss 780 inverted confocal microscope. Image fluorescence analysis was performed with the NIH Image J software.

### Real-Time PCR

Total RNA was isolated using the RNA purification kit from IBI Scientific (Peosta, IA, USA). The TaqMan^®^ Reverse Transcriptase kit (Applied Biosystems, Carlsbad, CA, USA) was used to generate complementary DNA from 500 ng of RNA template as per the manufacturer’s instructions. Real-time PCR was performed using SYBR Green Supermix (Applied Biosystems). All amplification reactions were performed in triplicate, and GAPDH was used as internal control. The primer sequences used for real-time PCR are shown in Table S1 in Supplementary Material.

### Western Blot

Western blotting was performed as previously described using the Odyssey CLx Infrared Imaging System (Li-Cor Biosciences) (30). The membranes were probed with the following primary antibodies: phospho-PKCδ (tyr^311^), total PKCδ, and GAPDH. Phospho-PKCδ (tyr^311^) was obtained from Cell Signaling Technology (Danvers, MA, USA). Total PKCδ and anti-GAPDH antibodies were procured from Santa Cruz Biotechnology (Dallas, TX, USA). The IRDye-conjugated secondary antibodies (Li-Cor Biosciences) were used to detect the primary antibodies.

### Reactive Oxygen Species (ROS) Measurement

#### L-012 Chemiluminescence

L-012 (400 μM, Wako Chemicals), a luminol-based chemiluminescent probe, was used to determine ${\text{O}}_{2}^{{\;}^{•-}}$ generation as described previously (15). Fifty thousand CD11c^+^ BMiDCs were plated in white flat bottom 96-well microplates in sterile PBS and superoxide production by stimulated by PMA (1 µM). The chemiluminescence signal was measured every 2 min for 2 h at 37°C using a Clariostar Monochromator Microplate Reader (BMG Labtech, Cary, NC, USA). The specificity of L-012 for ${\text{O}}_{2}^{{\;}^{•-}}$ was confirmed by the addition of SOD (150 U/mL).

#### Dihydroethidium

CD11c^+^ BMiDCs were plated in 24-well plates and preincubated with 5 µM DHE for 30 min at 37°C. Cells were treated with vehicle or PMA (1 µM) for 30 min. The reaction was stopped by placing the culture plate on ice. The cells were washed twice with ice-cold PBS, fixed in 2% PFA, and fluorescence (excitation/emission: 518 nm/605 nm) was measured using flow cytometry.

### Maturation of DCs

Dendritic cells were treated with vehicle + unlabeled OVA (50 µg/mL), PMA (1 µM) + OVA, or PMA alone for 24 h. DCs were then washed twice with ice-cold PBS and stained with PE/Cy7-CD11c, FITC-MHC II, and APC-CD86 monoclonal antibodies or respective isotype controls for 30 min at 4°C. FACS analysis was performed to determine CD86 and MHCII expression in CD11c^+^ iDCs.

### Proinflammatory Cytokine Gene Expression and Cytokine Secretion

CD11c^+^ BMiDCs/splenic iDCs were incubated with vehicle or treated with PMA in the presence or absence of OVA for 24 h. Cells were harvested and used to determine mRNA levels of various cytokines using real-time PCR. Cytokine secretion into the media was quantified using the LEGENDplex™ (mouse inflammation panel) bead-based immunoassay (BioLegend, San Diego, CA, USA) according to the manufacturer’s instructions. Data were acquired using four-color BD FACSCalibur (BD Biosciences, San Jose, CA, USA) and analyzed using the LEGENDplex Data Analysis software (BioLegend).

### Statistical Analysis

The data are expressed as mean ± SD. Statistical analysis was performed using GraphPad Prism (La Jolla, CA, USA). Student’s *t*-test and one- or two-way ANOVA, followed by a Bonferroni *post hoc* test, were used as appropriate for the particular experiment and treatment groups. A *p*-value less than 0.05 was considered statistically significant.

## Results

### PKC Promotes Macropinocytosis of Antigens in Stimulated iDCs

**Figure 1:**
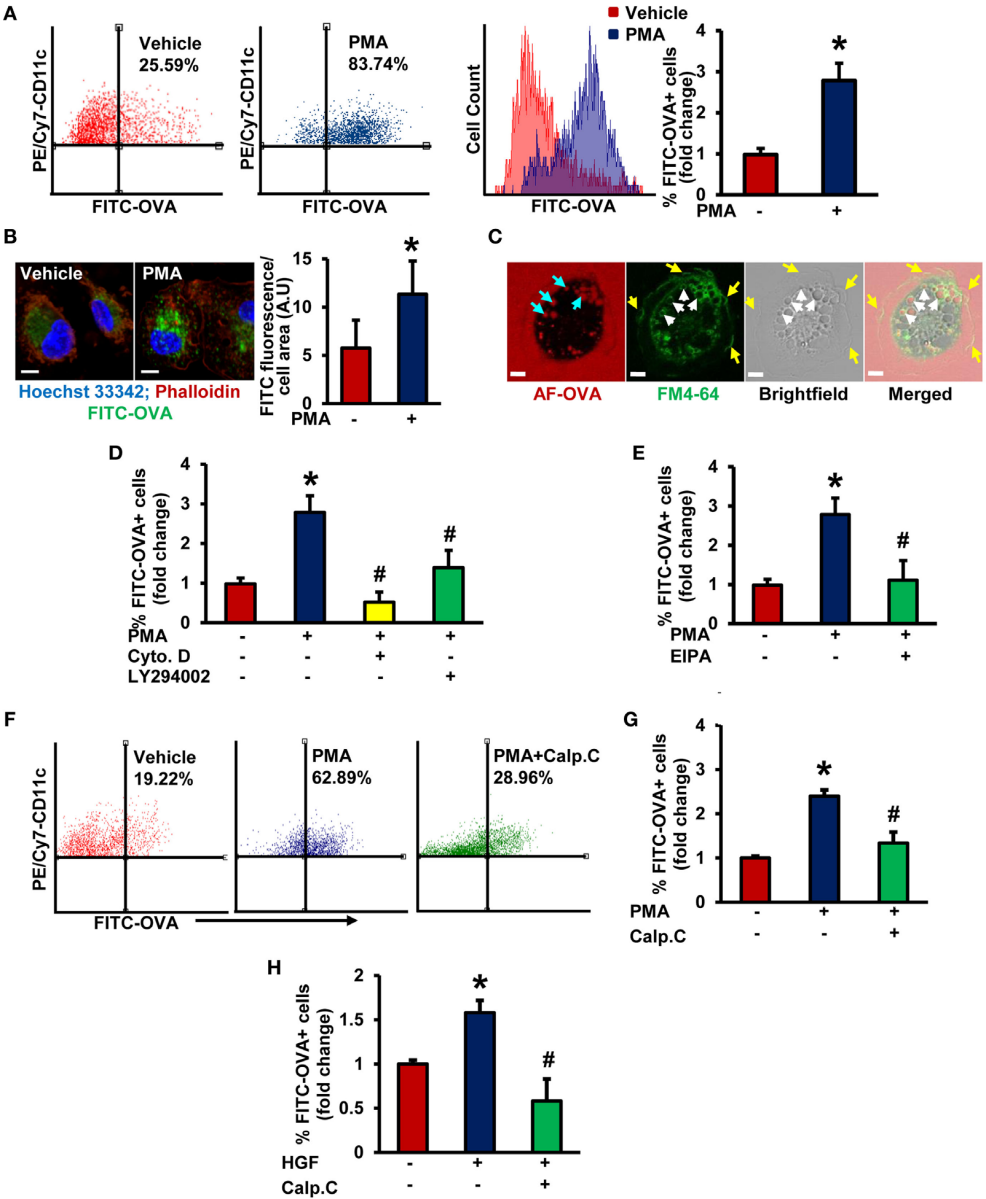
Protein kinase C activation promotes macropinocytosis of antigens in stimulated immature DCs (iDCs). **(A)** Wild-type (WT) bone marrow-derived immature dendritic cells (BMiDCs) were incubated with FITC-OVA (50 µg/mL; 45,000 MW) and treated with or without phorbol 12-myristate 13-acetate (PMA, 1 µM) for 5 h. Cells were stained with PE/Cy7-conjugated CD11c (or respective isotype control) monoclonal antibodies, fixed in 2% paraformaldehyde, and FITC fluorescence analyzed in PE/Cy7-gated CD11c^+^ cells using a Becton Dickinson FACSCalibur flow cytometer. Gating strategy for flow cytometry analysis is shown in Figure S1 in Supplementary Material. Bar diagram shows the mean fold change in percentage of FITC fluorescence^+^ cells among CD11c^+^ population (*n* = 7). **(B)** WT CD11c^+^ BMiDC were incubated with FITC-OVA (50 µg/mL, green) and stimulated with vehicle or PMA (1 µM) for 5 h. Cells were fixed in 2% PFA, and stained with TRITC-phalloidin (red) and Hoechst 33342 (blue). Images were taken using a Zeiss 780 inverted confocal microscope (63×). Similar findings have been observed in three independent experiments. Scale bar: 5 µm. Bar diagram represents quantified mean FITC fluorescence normalized to cell area in vehicle- and PMA-treated cells. **(C)** WT CD11c^+^ BMiDCs were treated with PMA (1 µM) for 30 min and incubated with the plasma membrane dye FM™ 4-64 (green) and Alexa Fluor 488-OVA (AF-OVA, 50 µg/mL, red) for 10 min. Live cell imaging was performed using a Zeiss 780 inverted confocal microscope. Yellow arrows indicate membrane ruffles, white arrows indicate membrane-derived macropinosomes, and blue arrows point to internalized extracellular solutes in macropinosomes. Images are representative of three independent experiments. Scale bar: 5 µm. **(D)** WT BMiDC were preincubated with vehicle, cytochalasin D (Cyto. D, 1 µM, 30 min) or LY294002 (10 µM, 30 min), treated with PMA (1 µM), and incubated with FITC-OVA for 5 h. FITC-OVA uptake was analyzed by fluorescence-activated cell sorting (FACS) (*n* = 3). **(E)** WT BMiDC were preincubated with vehicle or EIPA (25 µM) for 30 min, and challenged with PMA (1 µM, 5 h) in the presence of FITC-OVA. FITC-OVA internalization was analyzed by FACS (*n* = 5). **(F,G)** WT BMiDC were preincubated with vehicle or calphostin C (Calp. C, 100 nM, 30 min), treated with PMA, and FACS was performed as described above (*n* = 3). **(H)** WT BMiDC were preincubated with vehicle or calphostin C (Calp. C, 100 nM, 30 min), treated with HGF (100 ng/mL) in the presence of FITC-OVA for 5 h and FACS was performed to analyze FITC-OVA internalization. Bar diagram represents the mean fold change in FITC fluorescence^+^ cells (*n* = 3). Data represent the mean ± SD. *p < 0.05 vs. vehicle, ^#^*p* < 0.05 vs. PMA/HGF.

Although previous studies demonstrated that DCs use macropinocytosis to internalize antigens (5, 31), the signaling mechanisms involved remain poorly understood. iDCs were characterized by high-level expression of CD11c (CD11c^+^), low- to intermediate-level expression of MHC II, and low-level expression of CD86 (Figures S1 and S2 in Supplementary Material). LPS from *Escherichia coli*, a known DC maturation agent, was used as a positive control to determine the maturation status of DCs. FACS analysis demonstrated that PMA treatment stimulated internalization of fluorescent OVA in wild-type (WT) BMiDCs compared to vehicle-treated controls (Figure 1A). Gating strategy for FACS analysis is shown in Figure S1 in Supplementary Material. PMA-induced internalization of OVA was confirmed by confocal laser scanning microscopy (Figure 1B). Membrane ruffle formation and internalized Alexa Fluor 488-OVA in membrane-derived macropinosomes (~2–3 µm in diameter) following PMA treatment indicate macropinocytosis as the endocytic mechanism responsible for antigen uptake (Figure 1C). Macropinocytosis is pharmacologically characterized by its sensitivity to inhibitors of actin polymerization (32, 33), PI3K (30, 34), and sodium–hydrogen (Na^+^/H^+^) exchangers (15, 35). As shown in Figures 1D,E and Figure S3 in Supplementary Material, preincubation of BMiDCs with the actin perturbant, cytochalasin D (1 µM, 30 min), PI3K inhibitor, LY294002 (10 µM, 30 min), and Na^+^/H^+^ blocker, EIPA (25 µM, 30 min) completely blocked PMA-induced antigen internalization. Taken together, these data demonstrate that PMA stimulates internalization of antigens in DCs *via* macropinocytosis. Next, we investigated the role of PKC in DC macropinocytosis. BMiDCs were preincubated with vehicle or calphostin C (100 nM, 30 min), an inhibitor of classical and novel PKC isoforms (36), and macropinocytosis was stimulated using PMA. As shown in Figures 1F,G, calphostin C pretreatment inhibited PMA-stimulated macropinocytosis. To confirm the physiological importance of PKC in macropinocytosis, BMiDCs were incubated with HGF (100 ng/mL, 5 h), a physiologically relevant stimulator of macropinocytosis (13) and OVA uptake was analyzed by FACS analysis. HGF-stimulated OVA internalization in BMiDCs was inhibited by calphostin C pretreatment (100 nM, 30 min) (Figure 1H). These data demonstrate that activation of PKC in iDCs is required for stimulated macropinocytosis of OVA.

### PKCδ Activation Plays a Critical Role in Macropinocytosis of Antigens by iDCs

**Figure 2:**
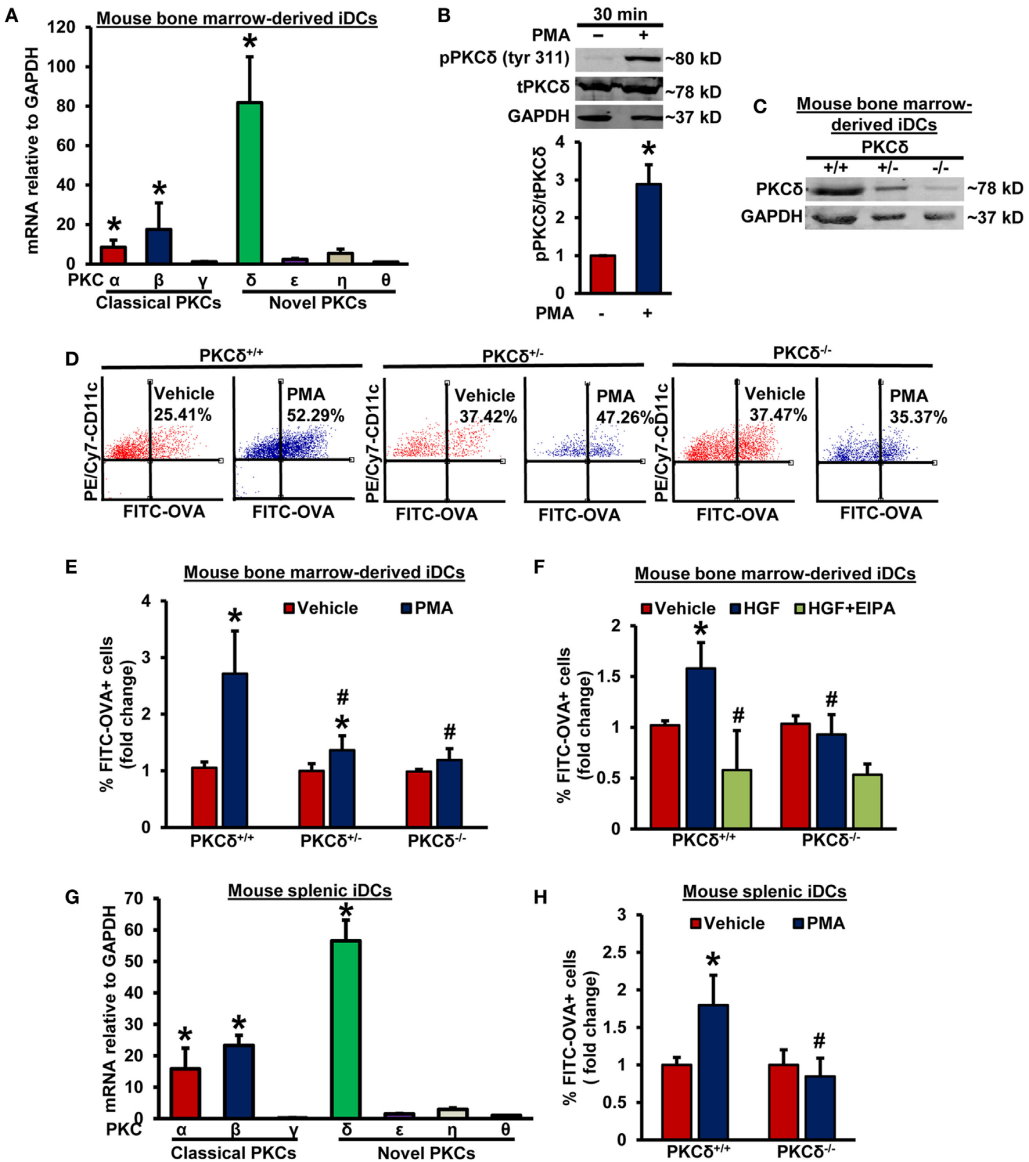
PKCδ activation mediates macropinocytosis of antigens by iDCs. **(A)** WT CD11c^+^ BMiDCs were subjected to qRT-PCR to determine mRNA expression of classical and novel protein kinase C (PKC) isoforms. GAPDH was used as an internal control. The transcript levels were calculated using delta–delta-CT method. Bar graph represents mRNA levels of classical and novel PKC isoforms in comparison to PKCθ (gene with lowest expression). Data represent the mean ± SEM. *p < 0.05 vs. PKCθ. **(B)** WT BMiDCs were treated with vehicle or PMA (1 µM) for 30 min. Cell lysates were used for Western blot analysis of phospho- PKCδ (tyr311), total PKCδ, and GAPDH protein expression. Representative Western blot images are shown. Bar graph represents averaged protein levels determined using densitometric analysis and expressed as a ratio of phospho- to total PKCδ (*n* = 6). **(C)** CD11c^+^ BMiDCs from three PKCδ^+/+^, PKCδ^+/−^, and PKCδ^−/−^ mice were pooled and Western blot was performed with cell lysates to validate deletion of PKCδ in DCs. **(D)** PKCδ^+/+^, PKCδ^+/−^, and PKCδ^−/−^ BMiDC were incubated with FITC-OVA (50 µg/mL) with or without PMA (1 µM) for 5 h. FITC fluorescence was analyzed using a Becton Dickinson FACSCalibur flow cytometer. **(E)** Bar diagram shows the fold change in mean FITC fluorescence^+^ cells among CD11c^+^ PKCδ^+/+^, PKCδ^+/−^, and PKCδ^−/−^ DCs (*n* = 6). **(F)** PKCδ^+/+^ and PKCδ^−/−^ BMiDC were preincubated for 30 min with EIPA (25 µM), and treated with hepatocyte growth factor (HGF, 100 ng/mL) for 5 h in the presence of FITC-OVA. FITC-OVA internalization was analyzed by fluorescence-activated cell sorting (FACS) (*n* = 4). **(G)** WT CD11c^+^ splenic iDCs were subjected to qRT-PCR for determination of various PKC isoforms. GAPDH was used as an internal control. Bar graph represents mRNA levels of PKC isoforms in comparison to PKCθ (gene with lowest expression). Data represent the mean ± SEM. *p < 0.05 vs. PKCθ. **(H)** Splenic iDCs from PKCδ^+/+^ and PKCδ^−/−^ mice were treated as described in Figure 2D for 1 h, and FITC fluorescence analyzed using FACS (*n* = 3). Data represent the mean ± SD. *p < 0.05 vs. vehicle, and ^#^*p* < 0.05 vs. PKCδ^+/+^ PMA/HGF.

To identify the potential PKC isoform(s) involved in DC macropinocytosis, we first examined mRNA expression of classical and novel PKC isoforms in WT CD11c^+^ BMiDCs using real-time PCR. As shown in Figure 2A, the DAG-dependent novel PKC isozyme, PKCδ, is the dominant PKC isoform in BMiDCs. Western blot results show that incubation of BMiDCs with PMA (1 µM, 30 min) activates PKCδ as demonstrated by its increased Tyr^311^ phosphorylation (Figure 2B) (37). To determine the functional role of PKCδ in DC macropinocytosis, we evaluated the ability of BMiDCs derived from WT PKCδ^+/+^ controls, heterozygous PKCδ^+/−^, and homozygous PKCδ^−/−^ mice to internalize antigens following PMA stimulation. The genotype of mice was confirmed by PCR analysis of genomic DNA (Figure S4 in Supplementary Material). PKCδ deletion in BMiDCs was confirmed by Western blotting (Figure 2C). FACS data demonstrated that PMA-induced antigen internalization is significantly attenuated in both PKCδ^+/−^ and PKCδ^−/−^ BMiDCs compared to PKCδ^+/+^ controls (Figures 2D,E). To test the physiological relevance of PKCδ in antigen macropinocytosis in iDCs, PKCδ^+/+^ and PKCδ^−/−^ BMiDCs were incubated with HGF (100 ng/mL, 5 h) and FITC-OVA internalization was investigated. HGF-stimulated internalization of FITC-OVA in PKCδ^+/+^ iDCs, however, we observed no stimulation in PKCδ^−/−^ iDCs (Figure 2F). The macropinocytosis inhibitor EIPA (25 µM, 30 min) abolished HGF-induced FITC-OVA uptake in PKCδ^+/+^ iDCs confirming the uptake mechanism as macropinocytosis. Consistent with our results in BMiDCs, RT-PCR experiments demonstrate that PKCδ is the dominant PKC isoform expressed in wild-type CD11c^+^ splenic and FLT3L-differentiated BM-derived DCs (Figure 2G; Figure S5A in Supplementary Material). Similar to BMiDCs, PMA stimulated FITC-OVA internalization in CD11c^+^ PKCδ^+/+^ splenic iDCs, but not in PKCδ^−/−^ splenic iDCs (Figure 2H). Taken together, these results suggest for the first time that stimulated iDC macropinocytosis involves PKCδ activation.

### Stimulation of Nox2 Activity Facilitates Macropinocytosis of Antigens in iDCs

**Figure 3:**
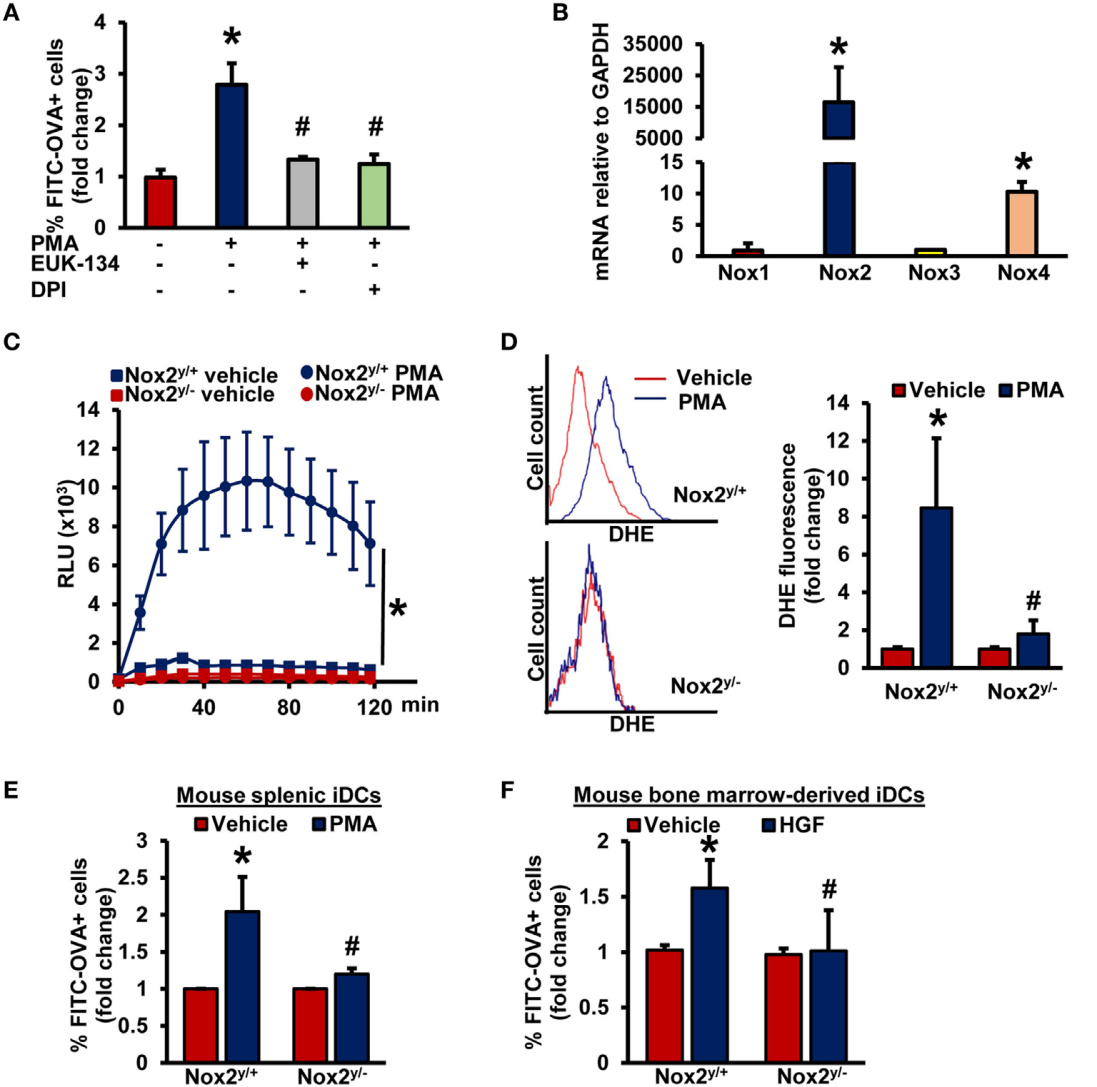
Nox2 activation induces macropinocytosis of antigens in iDCs. **(A)** WT BMiDCs were preincubated with EUK-134 (10 µM) or DPI (10 µM) for 30 min, and treated with PMA (1 µM) in the presence of FITC-OVA for 5 h. Percentage of CD11c^+^ FITC-OVA^+^ cells was analyzed by fluorescence-activated cell sorting (FACS) (*n* = 3). **(B)** qRT-PCR was used to determine mRNA expression of Nox1, Nox2, Nox3, and Nox4 in iDCs. GAPDH was used as an internal control. Data are representative of three independent experiments done in triplicate. **(C)** Nox2^y/+^ and Nox2^y/−^ BMiDCs were treated with vehicle or PMA (1 µM) and ${\text{O}}_{2}^{{\;}^{•-}}$ production was monitored using L-012 chemiluminescence (*n* = 3). **(D)** Intracellular ${\text{O}}_{2}^{{\;}^{•-}}$ production in vehicle and PMA-treated Nox2^y/+^ and Nox2^y/−^ BMiDCs was measured using dihydroethidium (DHE, 5 µM, 30 min.). Dihydroethidium (DHE) fluorescence was determined using fluorescence-activated cell sorting (FACS) analysis. Representative FACS histograms and bar graph are shown (*n* = 3). **(E,F)** Nox2^y/+^ and Nox2^y/−^ splenic **(E)** and BMiDCs **(F)** were incubated with vehicle, PMA, or HGF in the presence of FITC-OVA. FITC fluorescence was analyzed using FACS (*n* = 4). Data represent the mean ± SD. *p < 0.05 vs. vehicle and ^#^*p* < 0.05 vs. Nox2^y/+^ PMA/HGF.

As stimulation of PKC with phorbol esters and growth factors phosphorylates the Nox organizer subunit p47*^phox^*, leading to Nox2 activation and subsequent ${\text{O}}_{2}^{{\;}^{•-}}$ generation (26, 27, 38, 39), we postulated that (a) ${\text{O}}_{2}^{{\;}^{•-}}$ is involved as a signaling molecule in iDC macropinocytosis and (b) Nox2 activation contributes to antigen macropinocytosis by iDCs. Interestingly, a recent study by our laboratory has demonstrated the role of Nox2 in macrophage macropinocytosis of native LDL, leading to lipid accumulation and foam cell formation (15). To our knowledge, no prior studies have investigated the role of Nox enzymes and ${\text{O}}_{2}^{{\;}^{•-}}$ generation in DC macropinocytosis. Pretreatment of BMiDCs with diphenyleneiodonium (DPI; 10 µM, 30 min), a flavoenzyme inhibitor of Nox enzymes and other oxidases, and EUK-134 (10 µM, 30 min), a cell-permeable ${\text{O}}_{2}^{{\;}^{•-}}$ scavenger, inhibited PMA-induced antigen macropinocytosis (Figure 3A). As shown in Figure 3B, Figures S5B,C in Supplementary Material, Nox2 is the most highly expressed Nox isoform in GM-CSF/IL-4- and FLT3L-differentiated murine BMiDCs and splenic iDCs [Nox5 is not expressed in rodents (40)]. No significant difference was observed in Nox2 expression between PKCδ^+/+^ and PKCδ^−/−^ BMiDCs (Figure S5D in Supplementary Material). Control experiments also demonstrated that PKCδ expression in Nox2^y/+^ and Nox2^y/−^ BMiDCs is not different (Figure S5E in Supplementary Material). L-012 chemiluminescence and DHE fluorescence assays demonstrate that treatment of WT (Nox2^y/+^) iDCs with PMA stimulates ${\text{O}}_{2}^{{\;}^{•-}}$ generation, but there was no induction in Nox2^y/−^ iDCs (Figures 3C,D; Figure S6 in Supplementary Material). Furthermore, time-course L-012 chemiluminescence experiments demonstrate that Nox2-derived ${\text{O}}_{2}^{{\;}^{•-}}$ generation precedes or occurs concomitantly with PMA-induced PKCδ Tyr^311^ phosphorylation in iDCs (Figures S7A,B in Supplementary Material). The specificity of L-012 for ${\text{O}}_{2}^{{\;}^{•-}}$ was confirmed by the addition of SOD (150 U/ml) (not shown). The functional role of PKCδ in PMA-induced ${\text{O}}_{2}^{{\;}^{•-}}$ generation is demonstrated in Figure S7C in Supplementary Material.

To investigate the role of Nox2 in DC macropinocytosis of antigens, we treated splenic iDCs and BMiDCs isolated from WT and Nox2 knockout mice with fluorescently labeled OVA, stimulated cells with PMA and analyzed fluorescence using FACS. As shown in Figure 3E and Figure S8 in Supplementary Material, PMA-induced antigen accumulation was significantly attenuated in both Nox2^y/−^ splenic and BM-derived iDCs compared to WT controls. Finally, loss of Nox2 in BMiDCs inhibited HGF-induced macropinocytosis of antigens (Figure 3F). Taken together, these observations suggest that the PKCδ/Nox2/${\text{O}}_{2}^{{\;}^{•-}}$ signaling pathway plays an important role in antigen macropinocytosis by iDCs.

### PKCδ- and Nox2-Mediated Antigen Macropinocytosis Facilitates Maturation of iDCs

**Figure 4:**
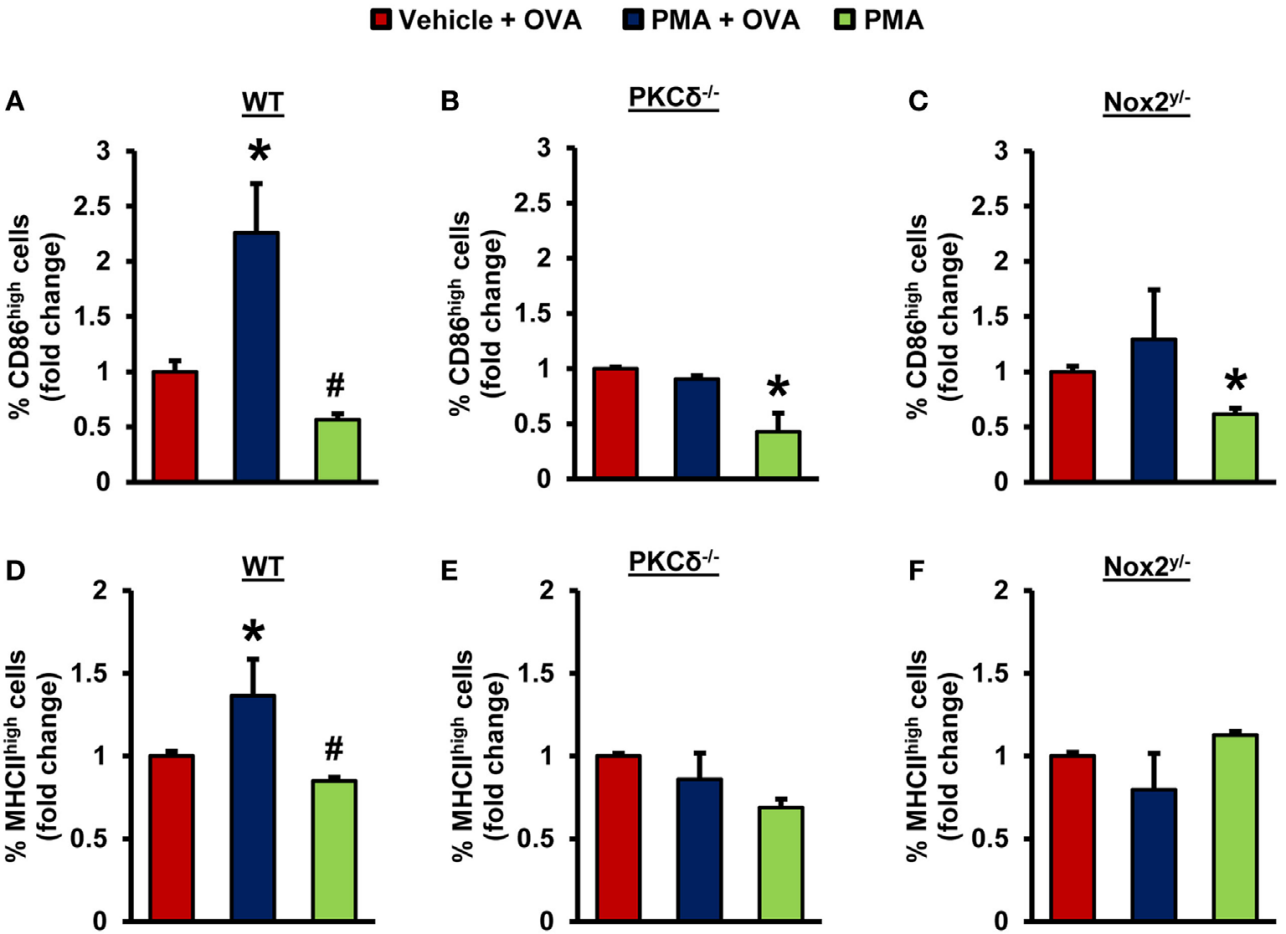
PKCδ- and Nox2-mediated antigen macropinocytosis stimulates maturation of iDCs. WT (C57BL/6J), PKCδ^−/−^, and Nox2^y/−^ BMiDCs were treated with vehicle + unlabeled OVA (50 µg/mL), PMA (1 µM) + OVA, or PMA alone for 24 h. FACS analysis was performed to determine CD86 **(A–C)** and MHCII **(D–F)** expression in CD11c^+^ iDCs. Bar graphs show fold changes in the percentage of CD86^high^/MHCII^high^ cells compared to “vehicle + OVA” treatment (*n* = 3). Data represent the mean ± SD. *p < 0.05 vs. vehicle + OVA, ^#^*p* < 0.05 vs. PMA + OVA.

Immature DCs internalize antigens and present the processed antigenic peptides loaded on MHC II molecules to naïve T-cells in lymphoid organs to initiate adaptive immune responses (41, 42). During this process, iDCs acquire a phenotype of mature DCs, which includes translocation of MHC II from intracellular endocytic compartments to the plasma membrane and increased surface expression of costimulatory molecules, such as CD86 (43). Next, we sought to investigate whether PKCδ–Nox2-mediated macropinocytosis of antigens by iDCs stimulates their maturation into mature and antigen-presenting DCs. PMA treatment in the presence of OVA stimulated plasma membrane expression of MHC II and CD86 in WT DCs, demonstrating that antigen macropinocytosis induces DC maturation (Figures 4A,D). Importantly, the results of our FACS analysis also indicate that PMA in the presence of OVA did not stimulate MHC II and CD86 plasma membrane expression in PKCδ^−/−^ and Nox2^y/−^ DCs (Figures 4B,C,E,F). Altogether, these data suggest that iDCs derived from PKCδ^−/−^ and Nox2^y/−^ mice have reduced maturation and antigen-presenting capacity due to their attenuated ability to internalize antigens *via* macropinocytosis.

### PKCδ- and Nox2-Mediated Antigen Macropinocytosis Plays an Important Role in DC Secretion of T-Cell-Regulatory Cytokines

**Figure 5:**
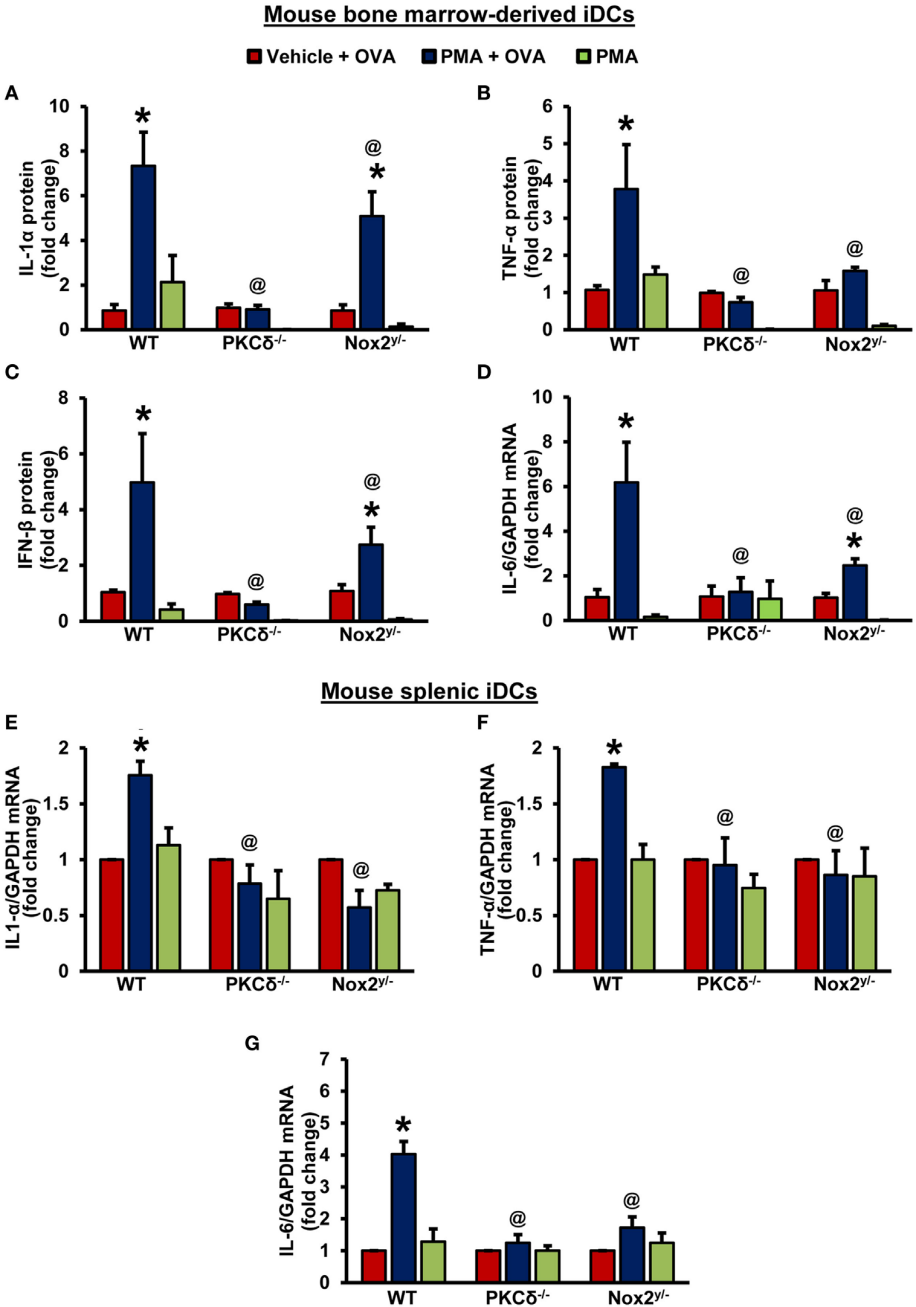
PKCδ- and Nox2-mediated antigen macropinocytosis regulates DC secretion of T-cell regulatory cytokines. **(A–C)** Wild-type (WT) (C57BL/6J), PKCδ^−/−^ and Nox2^y/−^ BMiDCs were treated with vehicle or PMA in the presence of unlabeled OVA (50 µg/mL) or PMA alone for 24 h. Cell culture supernatants were collected and levels of secreted cytokines determined using LEGENDplex™ bead-based immunoassay assay. Bar graphs show fold changes in cytokine secretion compared to “vehicle + OVA” treatment. Data are representative of three independent experiments and values are expressed as mean ± SD. **(D)** BMiDCs were treated as described above. The mRNA levels of IL-6 were determined by qRT-PCR. Bar graphs show fold change in the expression of IL-6 compared to “vehicle + OVA” treatment (*n* = 3) and data represent the mean ± SD. **(E–G)** WT, PKCδ^−/−^, and Nox2^y/−^ CD11c^+^ splenic iDCs were treated with vehicle + OVA, PMA + OVA, or PMA alone for 24 h. The mRNA levels of cytokines were determined by qRT-PCR. Data are representative of three independent experiments performed in triplicate and represent the mean ± SEM. Bar graphs show fold change in the expression compared to “vehicle + OVA” treatment. *p < 0.05 vs. vehicle + OVA, and *^@^p* < 0.05 vs. WT PMA + ova.

The antigen-specific T-cell receptor-mediated signaling, the co-stimulatory signals-mediated by DC CD80/CD86, and soluble cytokines secreted by DCs determine T-helper cell polarization and the functional consequences of antigen internalization (44). DCs secrete T_H_1-cell-polarizing factors, such as interleukin (IL)-12, interferons (IFNs) (44), TNF-α (45), and IL-27 (46). T_H_2 cell polarizing factors include monocytic chemotactic protein 1 (MCP1, also known as CCL2) and OX40 ligand (44); and IL-6, TGF-β, IL-1 (47), and IL-23 (48, 49) are known as T_H_17-cell-inducing cytokines. Hence, we next investigated the ability of DCs to produce various T-cell polarizing cytokines following PKCδ/Nox2-mediated antigen macropinocytosis. As shown in Figures 5A–C, OVA macropinocytosis stimulated secretion of IL-1α, TNF-α, and IFN-β proteins in WT iDCs. IL-1α, TNF-α, and IFN-β secretion by PKCδ^−/−^ and Nox2^y/−^ iDCs was significantly decreased compared to WT controls (Figures 5A–C). Similarly, IL-6 mRNA levels were stimulated by OVA macropinocytosis in WT, but not in PKCδ^−/−^ and Nox2^y/−^, iDCs (Figure 5D). Levels of IL-1β, IL-10, IL-12p70, IL-23, IL-27, IL-17A, and IFN-γ cytokines were also examined in cell culture supernatant, but we found either no stimulation of these cytokines following OVA macropinocytosis or were undetectable (data not shown). These changes in cytokines were also confirmed employing CD11c^+^ splenic DCs from WT, PKCδ^−/−^, and Nox2^y/−^ mice. As shown in Figures 5E–G PMA-stimulated OVA macropinocytosis induced mRNA expression of IL-6, TNF-α, and IL-1α in WT splenic DCs; however, there was no increase in cytokine levels in PKCδ^−/−^ and Nox2^y/−^ splenic DCs. Overall, these data demonstrate that PKCδ- and Nox2-mediated macropinocytosis of antigens plays an important role in DC secretion of T_H_1- and T_H_17-cell polarizing cytokines and may influence downstream T-cell function and signaling.

## Discussion

Dendritic cell macropinocytosis is a major mechanism of receptor-independent and indiscriminate sampling of extracellular proteins for antigen presentation (4, 5). Although our knowledge of the regulatory mechanisms in macropinocytosis has greatly increased in recent years (10, 31, 50), the precise signaling pathways responsible for the initiation and completion of DC macropinocytosis remain unknown. The present study demonstrates for the first time that PKCδ-mediated Nox2 activation stimulates DC macropinocytosis of antigens, leading to their maturation and secretion of specific inflammatory cytokines that may influence T-helper cell polarization and adaptive immunity. As such, these results may contribute to a better understanding of antigen macropinocytosis in DCs and downstream regulation of adaptive immune responses.

Recent studies by our lab and others have demonstrated that DAG-induced activation of PKC in macrophages stimulates macropinocytosis (15, 17). However, the role of PKC in DC macropinocytosis is currently unknown. The goal of the present study was to examine whether PKC activation stimulates DC macropinocytosis and, if so, to investigate the particular PKC isoform(s) involved and downstream signaling mechanisms leading to macropinocytosis. The members of PKC family are classified into three groups on the basis of their structure and cofactor requirements. *Classical PKCs* (α, β, and γ) are activated by DAG and Ca^2+^, *novel PKCs* (δ, ε, η, and θ) require only DAG for activation, while *atypical PKC isoforms* (λ, ι, μ, and ζ) are DAG-/Ca^2+^-independent enzymes (51, 52). Classical PKC isoforms contain tandem C1A/C1B motifs that bind DAG and a C2 domain that binds anionic phospholipids in a calcium-dependent manner. Novel PKCs also contain twin C1A/C1B domains and a C2 domain; however, the positions of the C1A/C1B and C2 domains are switched and novel PKC C2 domains do not bind calcium (53). Previous studies reported that growth factors and phorbol esters stimulate activation of DAG-dependent PKCs and peripheral actin reorganization (54, 55), suggesting a potential role for classical and/or novel PKC isoforms in DC macropinocytosis. To investigate the role of PKCs in DC macropinocytosis, we first pretreated iDCs with calphostin C, a known inhibitor of classical and novel PKCs, incubated cells with fluorescently labeled OVA and stimulated macropinocytosis. FACS data indicated that calphostin C inhibited both growth factor (HGF)- and phorbol ester-induced antigen internalization, suggesting a role of classical or novel PKC isoforms in DC macropinocytosis. The qRT-PCR experiments identified PKCδ as the dominant PKC isoform in FLT3L- and GM-CSF/IL-4-induced BMiDCs and splenic iDCs. Next, we investigated the role of PKCδ in DC macropinocytosis of OVA. Our results demonstrated that loss of PKCδ in iDCs completely inhibited OVA macropinocytosis in response to HGF- and PMA-treatments. These data indicate the role of novel PKC isoform, PKCδ as a signaling molecule in stimulated DC macropinocytosis. Consistent with our observations, previous studies demonstrated that rottlerin, which has been used as a PKCδ inhibitor, inhibit virus-induced macropinocytosis in endothelial cells (56) and attenuate parasite macropinocytosis by macrophages (57). It is important to add, however, that a number of studies questioned the selectivity of rottlerin as a PKCδ inhibitor and reported that it inhibits other kinases and non-kinase proteins and not PKCδ (58–60). Interestingly, previous findings also suggest a potential role of classical PKCs in macrophage and cancer cell macropinocytosis. Welliver and Swanson showed that PKCα levels are increased in macropinocytotic cups in macrophages following M-CSF stimulation (61). A more recent study by Yamamoto et al. reported that phorbol ester-induced macropinocytosis is inhibited in cancer cells expressing a mutant PKCγ isoform (62). We speculate that differences between these studies and our present work are related to the cell types used to study macropinocytosis, different PKC isoform expression, and differences in the mechanisms of macropinocytosis stimulation.

Phorbol esters mimic endogenously produced DAG and stimulate PKC activation, leading to a robust and long-lasting activation of Nox2 in phagocytes (24). Similarly, growth factors and M-CSF are known to stimulate Nox2-derived ${\text{O}}_{2}^{{\;}^{•-}}$ generation *via* PKC activation (15, 27, 39). Although these Nox2 activators are considered as the prototype stimulators of macropinocytosis, information regarding the role of Nox enzymes in DC macropinocytosis and downstream redox regulation of the multistep signaling process leading to macropinosome formation remains scant. To address this gap in knowledge, we investigated whether Nox-derived ROS in DCs contribute to stimulation of macropinocytosis in response to phorbol ester and growth factor treatments. The qRT-PCR data demonstrated that Nox2 is the major Nox isoform expressed in both BMiDCs (FLT3L- or GM-CSF/IL-4-induced) and *bonafide* splenic iDCs. Consistent with the role of Nox2 as the major source of ROS in DCs, two independent ${\text{O}}_{2}^{{\;}^{•-}}$ detection techniques (L-012 chemiluminescence and DHE fluorescence) demonstrated that PMA-induced ${\text{O}}_{2}^{{\;}^{•-}}$ production is significantly inhibited in Nox2 knockout iDCs compared to WT controls. It is important to note that increased DHE fluorescence in PMA-treated WT, but not in PKCδ^−/−^ or Nox2^y/−^ iDCs indicate that PKCδ-mediated Nox2 activation stimulates intracellular ${\text{O}}_{2}^{{\;}^{•-}}$ generation. Next, we demonstrated that DPI, a promiscuous inhibitor of flavin-containing oxidases, and EUK-134, a membrane-permeant ${\text{O}}_{2}^{{\;}^{•-}}$ scavenger, inhibited antigen uptake in iDCs, suggesting a potential role for Nox and intracellular ${\text{O}}_{2}^{{\;}^{•-}}$ signaling in macropinocytosis. To further extend these findings and to use a more specific approach, we incubated WT and Nox2 knockout splenic and bone marrow-derived iDCs with OVA and stimulated macropinocytosis with PMA and HGF. Our data demonstrated that both HGF- and PMA-induced macropinocytosis of fluorescently labeled OVA was significantly attenuated in Nox2^y/−^ iDCs compared with WT cells. To our knowledge, these results are the first to demonstrate the role of Nox2 in DC macropinocytosis. Nox2 enzymes expressed in internal membrane structures produce ROS intracellularly and play an important role as initiators and modulators of redox sensitive signaling pathways (63). Relevant to this point, we have recently demonstrated that Nox2 activation in macrophages stimulates intracellular ROS, leading to dephosphorylation of the actin-binding protein cofilin, membrane ruffling, and macropinocytosis (15). Interestingly, previous studies showed that endogenous ROS derived from Nox enzymes release slingshot phosphatase-1L (SSH-1L) from the inhibitory interaction with regulatory 14-3-3 proteins, leading to cofilin dephosphorylation and actin cytoskeleton reorganization (64). Based on these studies, we speculate that SSH-1L-mediated cofilin activation downstream of PKCδ/Nox2 signaling contributes to macropinocytosis stimulation. In addition to this potential mechanism, a number of other proteins involved in cytoskeletal reorganization are potential targets for oxidative modification and glutathiolation, including Src, Csk, actin, and protein tyrosine phosphatases that could be also playing a role in macropinocytosis (65).

Immature DCs after internalization of antigens undergo maturation and become professional APCs. During maturation, DCs lose their ability to endocytose antigens, increase surface expression of co-stimulatory molecules, such as CD86 and CD80, translocate MHCII to the plasma membrane, and change their morphology (66). We next investigated whether PKCδ/Nox2-mediated antigen macropinocytosis stimulates maturation of iDCs. Our results demonstrated that incubation of WT iDCs with PMA in the presence of OVA increased plasma membrane expression of CD86 and MHCII compared to PMA and OVA treatment alone, suggesting that macropinocytosis of antigens stimulates DC maturation. By contrast, PMA + OVA treatment did not stimulate plasma membrane expression of CD86 and MHCII in PKCδ and Nox2 knockout DCs. These findings indicate that iDCs derived from PKCδ^−/−^ and Nox2^y/−^ mice have decreased maturation capacity due to their reduced macropinocytic potential. Previous studies showed that pharmacological stimulation of PKC activity, and increased intracellular ROS production in the absence of antigens induce DC maturation and production of T-cell stimulatory cytokines (67–69). Consistent with these results, we found that PMA treatment alone (no OVA) stimulates IL-1α and TNF-α secretion in WT DCs (Figure S9 in Supplementary Material). New findings provided by the present study demonstrate that administration of PMA and OVA together significantly stimulates IL-1α and TNF-α secretion in WT DCs compared to PMA treatment alone. Based on these results, we propose that PKC–Nox2 signaling contributes to DC maturation through multiple mechanisms, including direct ${\text{O}}_{2}^{{\;}^{•-}}$-mediated signaling and stimulation of antigen macropinocytosis. It should also be noted that contrary to our observation a previous study found no stimulation of OVA internalization in DCs following PMA treatment (70). In this study, OVA was added 3 h after PMA treatment at which time point no stimulation of macropinocytosis is observed (15).

CD4^+^ T-cells play a major role in immune response and aid B cells to make antibodies, activate phagocytes, and recruit other immune cells to the site of infection/inflammation. Naïve CD4^+^ T-cells can differentiate into T_H_1, T_H_2, or T_H_17 cells, depending on pro-/anti-inflammatory cytokines present in the microenvironment (71). Mature DCs produce various cytokines and chemokines to orchestrate an efficient T_H_-cell response against infection (72). A variety of cytokines are produced by mature DCs, including IL-1, 6, 12 (73, 74), 23 (75), 27 (76, 77), TNF-α (73, 74), TGF-β (78), and IFNs (74), which play an important role in T_H_-cell polarization (44, 47). Macropinocytosis of OVA in WT BMiDCs stimulated secretion of IL-1α, TNF-α, and IFN-β and increased mRNA expression of IL-6. The observed increase in production of proinflammatory cytokines in WT cells were completely inhibited (IL-1α, TNF-α, IFN-β, and IL-6) or attenuated (IL-1α, IFN-β, and IL-6) in BMiDCs lacking PKCδ and Nox2, respectively. These findings suggest that IL-1α, IFN-β, and IL-6 secretion are mediated by Nox2-dependent and Nox2-independent pathways downstream of PKC. DC-derived TNF-α and IFNs have been shown to stimulate T_H_1 cell polarization and induce cytotoxic T lymphocyte-mediated immune responses (44, 79). Similarly, upregulation of transcript levels of IL-1α, TNF-α, and IL-6 were observed in WT splenic DCs and PKCδ/Nox2 deletion from these cells abrogated elevation of these cytokines. IL-1 and IL-6 promote differentiation and function of T_H_17 cells (80, 81). T_H_17 cells secrete IL-17 (IL-17A and IL-17F) cytokines, which are crucial for clearance of *Candida albicans* infection (82). Secretion of IL-17, IL-21, and IL-22 by T_H_17 cells correlates with the pathogenesis of several autoimmune (rheumatoid arthritis, systemic lupus erythematosus, multiple sclerosis, and psoriasis) and inflammatory diseases (inflammatory bowel disease and allergy and asthma) (83–89). The observed changes in the pattern of cytokine release and production suggest that PKCδ/Nox2-mediated antigen macropinocytosis may play a regulatory role in the development of specific T_H_1 and T_H_17 cells. In that respect, we expect future studies to investigate the role of DC antigen macropinocytosis in T-cell polarization, which is outside the scope of the present study.

In summary, our findings identified a previously unknown mechanism by which the novel PKC isoform, PKCδ *via* stimulation of Nox2 activity promotes macropinocytosis of antigens in DCs. The work presented herein shows that genetic blockade of PKCδ and Nox2 in DCs inhibit phorbol ester-stimulated macropinocytosis of antigens. Importantly, the role of PKCδ and Nox2 in DC macropinocytosis of antigens was also demonstrated in response to treatment with HGF, a physiologically relevant stimulator of macropinocytosis. PKCδ/Nox2-mediated antigen macropinocytosis stimulated DC maturation and induced secretion of various T_H_ cell regulatory cytokines. To our knowledge, these results are the first to describe redox regulation of macropinocytosis in DCs. As such, the present study may contribute to a better understanding of the regulatory mechanisms in DC macropinocytosis and downstream T-cell-mediated processes.

## Ethics Statement

All experimental procedures were approved by the Institutional Animal Care and Use Committee of Augusta University and conducted in accordance with the National Institutes of Health Guide for the Care and Use of Laboratory Animals.

## Author Contributions

Conception or design of the work: GC, BS, and PG; acquisition, data collection, and analysis: BS, PG, HL, and GC; writing and reviewing of manuscript: BS and GC. ZD provided PKCδ^+/−^ mice and QW helped with breeding and genotyping of animals. All authors approved the final version of the manuscript and agreed to be accountable for all aspects of the work in ensuring that questions related to the accuracy or integrity of any part of the work are appropriately investigated and resolved.

## Conflict of Interest Statement

The authors declare that the research was conducted in the absence of any commercial or financial relationships that could be taken as a potential conflict of interest.

## Supplementary Material

Figure S1 Gating strategy used for multicolor flow cytometry analysis. WT bone marrow-derived cells were incubated with FITC-OVA for 5 h, stained either with PE/Cy7-labeled CD11c **(B)** or respective IgG control **(A)** monoclonal antibodies, fixed in 2% paraformaldehyde, and processed for flow cytometry analysis. The cells were first gated on size and then doublets excluded using forward scatter area and forward scatter height. Cells were gated on CD11c^+^ population to identify dendritic cells (R1; green). Ovalbumin internalization was determined by measuring FITC fluorescence in R1 population (green) **(C)**.

Figure S2 Confirmation of immature phenotype of BMDCs. CD11c^+^ cells were incubated with vehicle or lipopolysaccharide (LPS) (500 ng/mL) for 24 h, fixed, and incubated with FITC-MHCII (clone M5/114.15.2), APC-CD86 (clone GL1) or respective IgG control monoclonal antibodies. CD86 **(A)** and MHC II **(B)** expression in CD11c^+^ dendritic cells were determined using flow cytometry. **p* < 0.05 vs. vehicle.

Figure S3 EIPA inhibits FITC-OVA uptake in bone marrow-derived immature dendritic cells (BMiDCs). **(A)** Wild-type (WT) CD11c^+^ BMiDCs were treated with vehicle or phorbol 12-myristate 13-acetate (PMA, 1 µM, 5 h), or pretreated with EIPA (25 µM, 30 min.) followed by PMA treatment in the presence of FITC-OVA. FITC-OVA internalization (green) was analyzed by confocal microscopy. Scale bar: 5 µm. **(B)** Bar diagram shows quantified FITC fluorescence intensity normalized to cell area (*n* = 3). At least five images per treatment were analyzed for each independent experiment using Image J. **p* < 0.05 vs. vehicle, *^#^p* < 0.05 vs. PMA.

Figure S4 Representative Agarose gel electrophoresis image showing mice genotyping for PKCδ expression.

Figure S5 PKCδ and Nox2 is the dominant PKC and Nox isoform in FLT3L-induced immature DCs (iDCs). **(A)** WT FLT3L-differentiated CD11c^+^ iDCs were subjected to qRT-PCR for determination of various PKCs levels. GAPDH was used as an internal control. Data are representative of three independent experiments performed in triplicate. Bar graph represents mRNA levels in comparison to PKCθ. **p* < 0.05 vs. PKCθ. **(B)** Transcript levels of Nox1, Nox2, Nox3, and Nox4 in WT FLT3L-differentiated CD11c^+^ iDCs. Bar graph represents mRNA levels in comparison to Nox1 (gene with lowest expression). **p* < 0.05 vs. Nox1. **(C)** Nox1, Nox2, Nox3, and Nox4 mRNA expression in WT CD11c^+^ splenic iDCs. **p* < 0.05 vs. Nox1. **(D)** PKCδ expression in Nox2^y/+^ and Nox2^y/−^ BMiDCs (*n* = 3). **(E)** Nox2 expression in PKCδ^+/+^ and PKCδ^−/−^ BMiDCs (*n* = 3). Data represent the mean ± SEM **(A–E)**.

Figure S6 Nox2 activation stimulates reactive oxygen species production in iDCs. Nox2^y/+^ (wild type) and Nox2^y/−^ BMiDCs were treated with vehicle or PMA (1 µM) and ${\text{O}}_{2}^{{\;}^{•-}}$ production was monitored using L-012 chemiluminescence. The diagram represents the mean fold change of L-012 chemiluminescence area under the curve obtained in three independent experiments. **p* < 0.05 vs. vehicle, ^#^*p* < 0.05 vs. Nox2^y/+^ PMA.

Figure S7 Stimulation of Nox2-derived ${\text{O}}_{2}^{{\;}^{•-}}$ generation in PMA-treated immature DCs precedes or occurs simultaneously with PKCδ activation. **(A)** ${\text{O}}_{2}^{{\;}^{•-}}$ generation was measured in vehicle- and PMA-treated Nox2^y/+^ (wild type) and Nox2^y/−^ BMiDCs using L-012 chemiluminescence (*n* = 3). **(B)** WT BMiDCs were treated with vehicle (30 min) or incubated with PMA (1 µM) for 5, 15, and 30 min. Cells were lysed and subjected to Western blot analysis for phospho-PKCδ (tyr^311^), total PKCδ, and GAPDH protein expression. Representative Western blot images are shown. The bar graph represents the ratio of phospho- to total PKCδ from three to six independent experiments. **(C)** Dihydroethidium (DHE) fluorescence was quantified in vehicle- and PMA (1 µM, 30 min.)-treated PKCδ^+/+^ and PKCδ^−/−^ BMiDCs using fluorescence-activated cell sorting analysis. Representative histograms showing DHE fluorescence are presented (left and middle panels). Bar diagram indicates fold change in mean fluorescence intensity (*n* = 3). **p* < 0.05 vs. vehicle, ^#^*p* < 0.05 vs. PKCδ^+/+^ PMA.

Figure S8 Loss of Nox2 in BMiDCS attenuates PMA-stimulated macropinocytosis of ovalbumin (OVA). Nox2^y/+^ and Nox2^y/−^ BMiDCs were incubated with vehicle or stimulated with PMA (1 µM, 5 h) in the presence of FITC-OVA. FITC fluorescence was analyzed in CD11c^+^ cells using fluorescence-activated cell sorting (FACS) (*n* = 3). **p* < 0.05 vs. vehicle, *^#^p* < 0.05 vs. Nox2^y/+^ PMA.

Figure S9 “PMA + OVA” treatment induces a more pronounced secretion of DC-derived T-cell regulatory cytokines compared to PMA treatment alone. **(A–B)** WT BMiDCs left untreated or treated with PMA or PMA + OVA for 24 h. Cell culture supernatants were collected and levels of secreted cytokines determined using the LEGENDplex™ bead-based immunoassay. **p* < 0.05 vs. vehicle, and *^@^p* < 0.05 vs. PMA. **(C–D)** WT, PKCδ^−/−^, and Nox2^y/−^ BMiDCs were left untreated or treated with PMA for 24 h. Cell culture supernatants used for cytokine estimation as described in **(A–B)**. Data are representative of three independent experiments and values are expressed as mean ± SD. **p* < 0.05 vs. vehicle and *^@^p* < 0.05 vs. WT PMA.

Table S1 List of primers used for mRNA quantitation using real-time-PCR.

## References

[1] Mellman I Steinman RM Dendritic cells: specialized and regulated antigen processing machines. Cell (2001) 106(3):255–8. 10.1016/S0092-8674(01)00449-4 11509172

[2] Trombetta ES Mellman I. Cell biology of antigen processing in vitro and in vivo. Annu Rev Immunol (2005) 23:975–1028. 10.1146/annurev.immunol.22.012703.104538 15771591

[3] Villadangos JA Schnorrer P. Intrinsic and cooperative antigen-presenting functions of dendritic-cell subsets in vivo. Nat Rev Immunol (2007) 7(7):543–55. 10.1038/nri2103 17589544

[4] Liu Z Roche PA. Macropinocytosis in phagocytes: regulation of MHC class-II-restricted antigen presentation in dendritic cells. Front Physiol (2015) 6:1. 10.3389/fphys.2015.00001 25688210 PMC4311620

[5] Norbury CC. Drinking a lot is good for dendritic cells. Immunology (2006) 117(4):443–51. 10.1111/j.1365-2567.2006.02335.x 16556257 PMC1782244

[6] Humeniuk P Dubiela P Hoffmann-Sommergruber K. Dendritic cells and their role in allergy: uptake, proteolytic processing and presentation of allergens. Int J Mol Sci (2017) 18(7):E1491. 10.3390/ijms18071491 28696399 PMC5535981

[7] Mercer J Helenius A. Virus entry by macropinocytosis. Nat Cell Biol (2009) 11(5):510–20. 10.1038/ncb0509-510 19404330

[8] Stuart LM Ezekowitz RA. Phagocytosis: elegant complexity. Immunity (2005) 22(5):539–50. 10.1016/j.immuni.2005.05.002 15894272

[9] Lim JP Gleeson PA. Macropinocytosis: an endocytic pathway for internalising large gulps. Immunol Cell Biol (2011) 89(8):836–43. 10.1038/icb.2011.20 21423264

[10] Bohdanowicz M Grinstein S. Role of phospholipids in endocytosis, phagocytosis, and macropinocytosis. Physiol Rev (2013) 93(1):69–106. 10.1152/physrev.00002.2012 23303906

[11] Roche PA Furuta K. The ins and outs of MHC class II-mediated antigen processing and presentation. Nat Rev Immunol (2015) 15(4):203–16. 10.1038/nri3818 25720354 PMC6314495

[12] Bryant DM Kerr MC Hammond LA Joseph SR Mostov KE Teasdale RD EGF induces macropinocytosis and SNX1-modulated recycling of E-cadherin. J Cell Sci (2007) 120(Pt 10):1818–28. 10.1242/jcs.000653 17502486

[13] Dowrick P Kenworthy P McCann B Warn R. Circular ruffle formation and closure lead to macropinocytosis in hepatocyte growth factor/scatter factor-treated cells. Eur J Cell Biol (1993) 61(1):44–53. 8223707

[14] BoseDasgupta S Moes S Jenoe P Pieters J Cytokine-induced macropinocytosis in macrophages is regulated by 14-3-3zeta through its interaction with serine-phosphorylated coronin 1. FEBS J (2015) 282(7):1167–81. 10.1111/febs.13214 25645340

[15] Ghoshal P Singla B Lin H Feck DM Cantu-Medellin N Kelley EE Nox2-mediated PI3K and cofilin activation confers alternate redox control of macrophage pinocytosis. Antioxid Redox Signal (2017) 26(16):902–16. 10.1089/ars.2016.6639 27488058 PMC5455614

[16] Swanson JA. Phorbol esters stimulate macropinocytosis and solute flow through macrophages. J Cell Sci (1989) 94(Pt 1):135–42. 2613767 10.1242/jcs.94.1.135

[17] Yoshida S Gaeta I Pacitto R Krienke L Alge O Gregorka B Differential signaling during macropinocytosis in response to M-CSF and PMA in macrophages. Front Physiol (2015) 6:8. 10.3389/fphys.2015.00008 25688212 PMC4310286

[18] Swanson JA. Shaping cups into phagosomes and macropinosomes. Nat Rev Mol Cell Biol (2008) 9(8):639–49. 10.1038/nrm2447 18612320 PMC2851551

[19] Nishikawa K Toker A Johannes FJ Songyang Z Cantley LC. Determination of the specific substrate sequence motifs of protein kinase C isozymes. J Biol Chem (1997) 272(2):952–60. 10.1074/jbc.272.2.952 8995387

[20] Sudan R Srivastava N Pandey SP Majumdar S Saha B. Reciprocal regulation of protein kinase C isoforms results in differential cellular responsiveness. J Immunol (2012) 188(5):2328–37. 10.4049/jimmunol.1101678 22271653

[21] Gong R Hong AW Plouffe SW Zhao B Liu G Yu FX Opposing roles of conventional and novel PKC isoforms in Hippo-YAP pathway regulation. Cell Res (2015) 25(8):985–8. 10.1038/cr.2015.88 26206313 PMC4528059

[22] Segal AW. The function of the NADPH oxidase of phagocytes and its relationship to other NOXs in plants, invertebrates, and mammals. Int J Biochem Cell Biol (2008) 40(4):604–18. 10.1016/j.biocel.2007.10.003 18036868 PMC2636181

[23] Bedard K Krause KH. The NOX family of ROS-generating NADPH oxidases: physiology and pathophysiology. Physiol Rev (2007) 87(1):245–313. 10.1152/physrev.00044.2005 17237347

[24] Inanami O Johnson JL McAdara JK Benna JE Faust LR Newburger PE Activation of the leukocyte NADPH oxidase by phorbol ester requires the phosphorylation of p47PHOX on serine 303 or 304. J Biol Chem (1998) 273(16):9539–43. 10.1074/jbc.273.16.9539 9545283

[25] Lavigne MC Malech HL Holland SM Leto TL. Genetic demonstration of p47phox-dependent superoxide anion production in murine vascular smooth muscle cells. Circulation (2001) 104(1):79–84. 10.1161/01.CIR.104.1.79 11435342

[26] Meijles DN Fan LM Howlin BJ Li JM. Molecular insights of p47phox phosphorylation dynamics in the regulation of NADPH oxidase activation and superoxide production. J Biol Chem (2014) 289(33):22759–70. 10.1074/jbc.M114.561159 24970888 PMC4132782

[27] Schroder K Schutz S Schloffel I Batz S Takac I Weissmann N Hepatocyte growth factor induces a proangiogenic phenotype and mobilizes endothelial progenitor cells by activating Nox2. Antioxid Redox Signal (2011) 15(4):915–23. 10.1089/ars.2010.3533 21050133

[28] Pierre P Turley SJ Gatti E Hull M Meltzer J Mirza A Developmental regulation of MHC class II transport in mouse dendritic cells. Nature (1997) 388(6644):787–92. 10.1038/42039 9285592

[29] Xu Y Zhan Y Lew AM Naik SH Kershaw MH. Differential development of murine dendritic cells by GM-CSF versus Flt3 ligand has implications for inflammation and trafficking. J Immunol (2007) 179(11):7577–84. 10.4049/jimmunol.179.11.7577 18025203

[30] Csanyi G Feck DM Ghoshal P Singla B Lin H Nagarajan S CD47 and Nox1 mediate dynamic fluid-phase macropinocytosis of native LDL. Antioxid Redox Signal (2017) 26(16):886–901. 10.1089/ars.2016.6834 27958762 PMC5455613

[31] Sandgren KJ Wilkinson J Miranda-Saksena M McInerney GM Byth-Wilson K Robinson PJ A differential role for macropinocytosis in mediating entry of the two forms of vaccinia virus into dendritic cells. PLoS Pathog (2010) 6(4):e1000866. 10.1371/journal.ppat.1000866 20421949 PMC2858709

[32] Oliveira CA Kashman Y Mantovani B. Effects of latrunculin A on immunological phagoctosis and macrophage spreading-associated changes in the F-actin/G-actin content of the cells. Chem Biol Interact (1996) 100(2):141–53. 10.1016/0009-2797(96)03695-2 8646787

[33] Aleksandrowicz P Marzi A Biedenkopf N Beimforde N Becker S Hoenen T Ebola virus enters host cells by macropinocytosis and clathrin-mediated endocytosis. J Infect Dis (2011) 204(Suppl 3):S957–67. 10.1093/infdis/jir326 21987776 PMC3189988

[34] Kruth HS Jones NL Huang W Zhao B Ishii I Chang J Macropinocytosis is the endocytic pathway that mediates macrophage foam cell formation with native low density lipoprotein. J Biol Chem (2005) 280(3):2352–60. 10.1074/jbc.M407167200 15533943

[35] Koivusalo M Welch C Hayashi H Scott CC Kim M Alexander T Amiloride inhibits macropinocytosis by lowering submembranous pH and preventing Rac1 and Cdc42 signaling. J Cell Biol (2010) 188(4):547–63. 10.1083/jcb.200908086 20156964 PMC2828922

[36] Larsen EC DiGennaro JA Saito N Mehta S Loegering DJ Mazurkiewicz JE Differential requirement for classic and novel PKC isoforms in respiratory burst and phagocytosis in RAW 264.7 cells. J Immunol (2000) 165(5):2809–17. 10.4049/jimmunol.165.5.2809 10946313

[37] Nakashima H Frank GD Shirai H Hinoki A Higuchi S Ohtsu H Novel role of protein kinase C-delta Tyr 311 phosphorylation in vascular smooth muscle cell hypertrophy by angiotensin II. Hypertension (2008) 51(2):232–8. 10.1161/HYPERTENSIONAHA.107.101253 18180404 PMC2728048

[38] Belambri SA Hurtado-Nedelec M Senator A Makni-Maalej K Fay M Gougerot-Pocidalo MA Phosphorylation of p47phox is required for receptor-mediated NADPH oxidase/NOX2 activation in Epstein-Barr virus-transformed human B lymphocytes. Am J Blood Res (2012) 2(3):187–93. 23119229 PMC3484414

[39] Heppner DE van der Vliet A. Redox-dependent regulation of epidermal growth factor receptor signaling. Redox Biol (2016) 8:24–7. 10.1016/j.redox.2015.12.002 26722841 PMC4710793

[40] Fulton DJ. Nox5 and the regulation of cellular function. Antioxid Redox Signal (2009) 11(10):2443–52. 10.1089/ARS.2009.2587 19331545 PMC2821136

[41] Winzler C Rovere P Rescigno M Granucci F Penna G Adorini L Maturation stages of mouse dendritic cells in growth factor-dependent long-term cultures. J Exp Med (1997) 185(2):317–28. 10.1084/jem.185.2.317 9016880 PMC2196118

[42] Steinman RM Pack M Inaba K. Dendritic cells in the T-cell areas of lymphoid organs. Immunol Rev (1997) 156:25–37. 10.1111/j.1600-065X.1997.tb00956.x 9176697

[43] Reis e Sousa C Dendritic cells in a mature age. Nat Rev Immunol (2006) 6(6):476–83. 10.1038/nri1845 16691244

[44] Kapsenberg ML Dendritic-cell control of pathogen-driven T-cell polarization. Nat Rev Immunol (2003) 3(12):984–93. 10.1038/nri1246 14647480

[45] Lutz MB Schuler G. Immature, semi-mature and fully mature dendritic cells: which signals induce tolerance or immunity? Trends Immunol (2002) 23(9):445–9. 10.1016/S1471-4906(02)02281-0 12200066

[46] Pflanz S Timans JC Cheung J Rosales R Kanzler H Gilbert J IL-27, a heterodimeric cytokine composed of EBI3 and p28 protein, induces proliferation of naive CD4+ T cells. Immunity (2002) 16(6):779–90. 10.1016/S1074-7613(02)00324-2 12121660

[47] DuPage M Bluestone JA. Harnessing the plasticity of CD4(+) T cells to treat immune-mediated disease. Nat Rev Immunol (2016) 16(3):149–63. 10.1038/nri.2015.18 26875830

[48] Aggarwal S Ghilardi N Xie MH de Sauvage FJ Gurney AL. Interleukin-23 promotes a distinct CD4 T cell activation state characterized by the production of interleukin-17. J Biol Chem (2003) 278(3):1910–4. 10.1074/jbc.M207577200 12417590

[49] Gee K Guzzo C Che Mat NF Ma W Kumar A. The IL-12 family of cytokines in infection, inflammation and autoimmune disorders. Inflamm Allergy Drug Targets (2009) 8(1):40–52. 10.2174/187152809787582507 19275692

[50] Sarkar K Kruhlak MJ Erlandsen SL Shaw S. Selective inhibition by rottlerin of macropinocytosis in monocyte-derived dendritic cells. Immunology (2005) 116(4):513–24. 10.1111/j.1365-2567.2005.02253.x 16313365 PMC1802442

[51] Parker PJ Murray-Rust J PKC at a glance. J Cell Sci (2004) 117(Pt 2):131–2. 10.1242/jcs.00982 14676268

[52] Newton AC. Protein kinase C: poised to signal. Am J Physiol Endocrinol Metab (2010) 298(3):E395–402. 10.1152/ajpendo.00477.2009 19934406 PMC2838521

[53] Steinberg SF Cardiac actions of protein kinase C isoforms. Physiology (Bethesda) (2012) 27(3):130–9. 10.1152/physiol.00009.2012 22689788 PMC3582339

[54] Arber S Barbayannis FA Hanser H Schneider C Stanyon CA Bernard O Regulation of actin dynamics through phosphorylation of cofilin by LIM-kinase. Nature (1998) 393(6687):805–9. 10.1038/31729 9655397

[55] Reyhani V Tsioumpekou M van Wieringen T Rask L Lennartsson J Rubin K PDGF-BB enhances collagen gel contraction through a PI3K-PLCgamma-PKC-cofilin pathway. Sci Rep (2017) 7(1):8924 10.1038/s41598-017-08411-1 28827622 PMC5566449

[56] Raghu H Sharma-Walia N Veettil MV Sadagopan S Chandran B. Kaposi’s sarcoma-associated herpesvirus utilizes an actin polymerization-dependent macropinocytic pathway to enter human dermal microvascular endothelial and human umbilical vein endothelial cells. J Virol (2009) 83(10):4895–911. 10.1128/JVI.02498-08 19279100 PMC2682094

[57] Barrias ES Reignault LC De Souza W Carvalho TM. Trypanosoma cruzi uses macropinocytosis as an additional entry pathway into mammalian host cell. Microbes Infect (2012) 14(14):1340–51. 10.1016/j.micinf.2012.08.003 23010292

[58] Soltoff SP. Rottlerin: an inappropriate and ineffective inhibitor of PKCdelta. Trends Pharmacol Sci (2007) 28(9):453–8. 10.1016/j.tips.2007.07.003 17692392

[59] Davies SP Reddy H Caivano M Cohen P. Specificity and mechanism of action of some commonly used protein kinase inhibitors. Biochem J (2000) 351(Pt 1):95–105. 10.1042/0264-6021:3510095 10998351 PMC1221339

[60] Gschwendt M Muller HJ Kielbassa K Zang R Kittstein W Rincke G Rottlerin, a novel protein kinase inhibitor. Biochem Biophys Res Commun (1994) 199(1):93–8. 10.1006/bbrc.1994.1199 8123051

[61] Welliver TP Swanson JA A growth factor signaling cascade confined to circular ruffles in macrophages. Biol Open (2012) 1(8):754–60. 10.1242/bio.20121784 23213469 PMC3507227

[62] Yamamoto K Seki T Yamamoto H Adachi N Tanaka S Hide I Deregulation of the actin cytoskeleton and macropinocytosis in response to phorbol ester by the mutant protein kinase C gamma that causes spinocerebellar ataxia type 14. Front Physiol (2014) 5:126. 10.3389/fphys.2014.00126 24744737 PMC3978357

[63] Brown DI Griendling KK. Nox proteins in signal transduction. Free Radic Biol Med (2009) 47(9):1239–53. 10.1016/j.freeradbiomed.2009.07.023 19628035 PMC2763943

[64] Zhou Y Yan H Guo M Zhu J Xiao Q Zhang L. Reactive oxygen species in vascular formation and development. Oxid Med Cell Longev (2013) 2013:374963. 10.1155/2013/374963 23401740 PMC3564431

[65] Valdivia A Duran C San Martin A. The role of Nox-mediated oxidation in the regulation of cytoskeletal dynamics. Curr Pharm Des (2015) 21(41):6009–22. 10.2174/1381612821666151029112624 26510432 PMC4699303

[66] Platt CD Ma JK Chalouni C Ebersold M Bou-Reslan H Carano RA Mature dendritic cells use endocytic receptors to capture and present antigens. Proc Natl Acad Sci U S A (2010) 107(9):4287–92. 10.1073/pnas.0910609107 20142498 PMC2840134

[67] Do Y Hegde VL Nagarkatti PS Nagarkatti M. Bryostatin-1 enhances the maturation and antigen-presenting ability of murine and human dendritic cells. Cancer Res (2004) 64(18):6756–65. 10.1158/0008-5472.CAN-03-4002 15374994

[68] Romero MM Basile JI Corra Feo L Lopez B Ritacco V Aleman M. Reactive oxygen species production by human dendritic cells involves TLR2 and dectin-1 and is essential for efficient immune response against Mycobacteria. Cell Microbiol (2016) 18(6):875–86. 10.1111/cmi.12562 26709456

[69] Matsue H Edelbaum D Shalhevet D Mizumoto N Yang C Mummert ME Generation and function of reactive oxygen species in dendritic cells during antigen presentation. J Immunol (2003) 171(6):3010–8. 10.4049/jimmunol.171.6.3010 12960326

[70] Majewski M Bose TO Sille FC Pollington AM Fiebiger E Boes M. Protein kinase C delta stimulates antigen presentation by Class II MHC in murine dendritic cells. Int Immunol (2007) 19(6):719–32. 10.1093/intimm/dxm034 17446207

[71] Kaiko GE Horvat JC Beagley KW Hansbro PM. Immunological decision-making: how does the immune system decide to mount a helper T-cell response? Immunology (2008) 123(3):326–38. 10.1111/j.1365-2567.2007.02719.x 17983439 PMC2433332

[72] Piemonti L Monti P Allavena P Sironi M Soldini L Leone BE Glucocorticoids affect human dendritic cell differentiation and maturation. J Immunol (1999) 162(11):6473–81. 10352262

[73] Dixon GL Newton PJ Chain BM Katz D Andersen SR Wong S Dendritic cell activation and cytokine production induced by group B *Neisseria meningitidis*: interleukin-12 production depends on lipopolysaccharide expression in intact bacteria. Infect Immun (2001) 69(7):4351–7. 10.1128/IAI.69.7.4351-4357.2001 11401973 PMC98506

[74] Morelli AE Zahorchak AF Larregina AT Colvin BL Logar AJ Takayama T Cytokine production by mouse myeloid dendritic cells in relation to differentiation and terminal maturation induced by lipopolysaccharide or CD40 ligation. Blood (2001) 98(5):1512–23. 10.1182/blood.V98.5.1512 11520802

[75] Roses RE Xu S Xu M Koldovsky U Koski G Czerniecki BJ. Differential production of IL-23 and IL-12 by myeloid-derived dendritic cells in response to TLR agonists. J Immunol (2008) 181(7):5120–7. 10.4049/jimmunol.181.7.5120 18802116

[76] Ziblat A Domaica CI Spallanzani RG Iraolagoitia XL Rossi LE Avila DE IL-27 stimulates human NK-cell effector functions and primes NK cells for IL-18 responsiveness. Eur J Immunol (2015) 45(1):192–202. 10.1002/eji.201444699 25308526

[77] Zhang X Tao Y Wang J Garcia-Mata R Markovic-Plese S. Simvastatin inhibits secretion of Th17-polarizing cytokines and antigen presentation by DCs in patients with relapsing remitting multiple sclerosis. Eur J Immunol (2013) 43(1):281–9. 10.1002/eji.201242566 23076801

[78] Jin Y Wi HJ Choi MH Hong ST Bae YM Regulation of anti-inflammatory cytokines IL-10 and TGF-beta in mouse dendritic cells through treatment with *Clonorchis sinensis* crude antigen. Exp Mol Med (2014) 46:e74 10.1038/emm.2013.144 24480801 PMC3909892

[79] Jordan SJ Gupta K Ogendi BM Bakshi RK Kapil R Press CG The predominant CD4(+) Th1 Cytokine Elicited to *Chlamydia trachomatis* infection in women is tumor necrosis factor alpha and not interferon gamma. Clin Vaccine Immunol (2017) 24(4):e10–7. 10.1128/CVI.00010-17 PMC5382828 28100498

[80] Sutton C Brereton C Keogh B Mills KH Lavelle EC. A crucial role for interleukin (IL)-1 in the induction of IL-17-producing T cells that mediate autoimmune encephalomyelitis. J Exp Med (2006) 203(7):1685–91. 10.1084/jem.20060285 16818675 PMC2118338

[81] Bettelli E Carrier Y Gao W Korn T Strom TB Oukka M Reciprocal developmental pathways for the generation of pathogenic effector TH17 and regulatory T cells. Nature (2006) 441(7090):235–8. 10.1038/nature04753 16648838

[82] Conti HR Shen F Nayyar N Stocum E Sun JN Lindemann MJ Th17 cells and IL-17 receptor signaling are essential for mucosal host defense against oral candidiasis. J Exp Med (2009) 206(2):299–311. 10.1084/jem.20081463 19204111 PMC2646568

[83] Zenewicz LA Antov A Flavell RA. CD4 T-cell differentiation and inflammatory bowel disease. Trends Mol Med (2009) 15(5):199–207. 10.1016/j.molmed.2009.03.002 19362058

[84] Maddur MS Miossec P Kaveri SV Bayry J. Th17 cells: biology, pathogenesis of autoimmune and inflammatory diseases, and therapeutic strategies. Am J Pathol (2012) 181(1):8–18. 10.1016/j.ajpath.2012.03.044 22640807

[85] Lubberts E Koenders MI van den Berg WB. The role of T-cell interleukin-17 in conducting destructive arthritis: lessons from animal models. Arthritis Res Ther (2005) 7(1):29–37. 10.1186/ar1478 15642151 PMC1064899

[86] Doreau A Belot A Bastid J Riche B Trescol-Biemont MC Ranchin B Interleukin 17 acts in synergy with B cell-activating factor to influence B cell biology and the pathophysiology of systemic lupus erythematosus. Nat Immunol (2009) 10(7):778–85. 10.1038/ni.1741 19483719

[87] Komiyama Y Nakae S Matsuki T Nambu A Ishigame H Kakuta S IL-17 plays an important role in the development of experimental autoimmune encephalomyelitis. J Immunol (2006) 177(1):566–73. 10.4049/jimmunol.177.1.566 16785554

[88] Ma HL Liang S Li J Napierata L Brown T Benoit S IL-22 is required for Th17 cell-mediated pathology in a mouse model of psoriasis-like skin inflammation. J Clin Invest (2008) 118(2):597–607. 10.1172/JCI33263 18202747 PMC2200300

[89] Cosmi L Maggi L Santarlasci V Capone M Cardilicchia E Frosali F Identification of a novel subset of human circulating memory CD4(+) T cells that produce both IL-17A and IL-4. J Allergy Clin Immunol (2010) 125(1):e1–4. 10.1016/j.jaci.2009.10.012 20109749

