# Supplementary figures and images for: PKCδ-Mediated Nox2 Activation Promotes Fluid-Phase Pinocytosis of Antigens by Immature Dendritic Cells
Source: Front Immunol. 2018 Mar 26;9:537. doi: 10.3389/fimmu.2018.00537 (PMC5879126; doi:10.3389/fimmu.2018.00537)

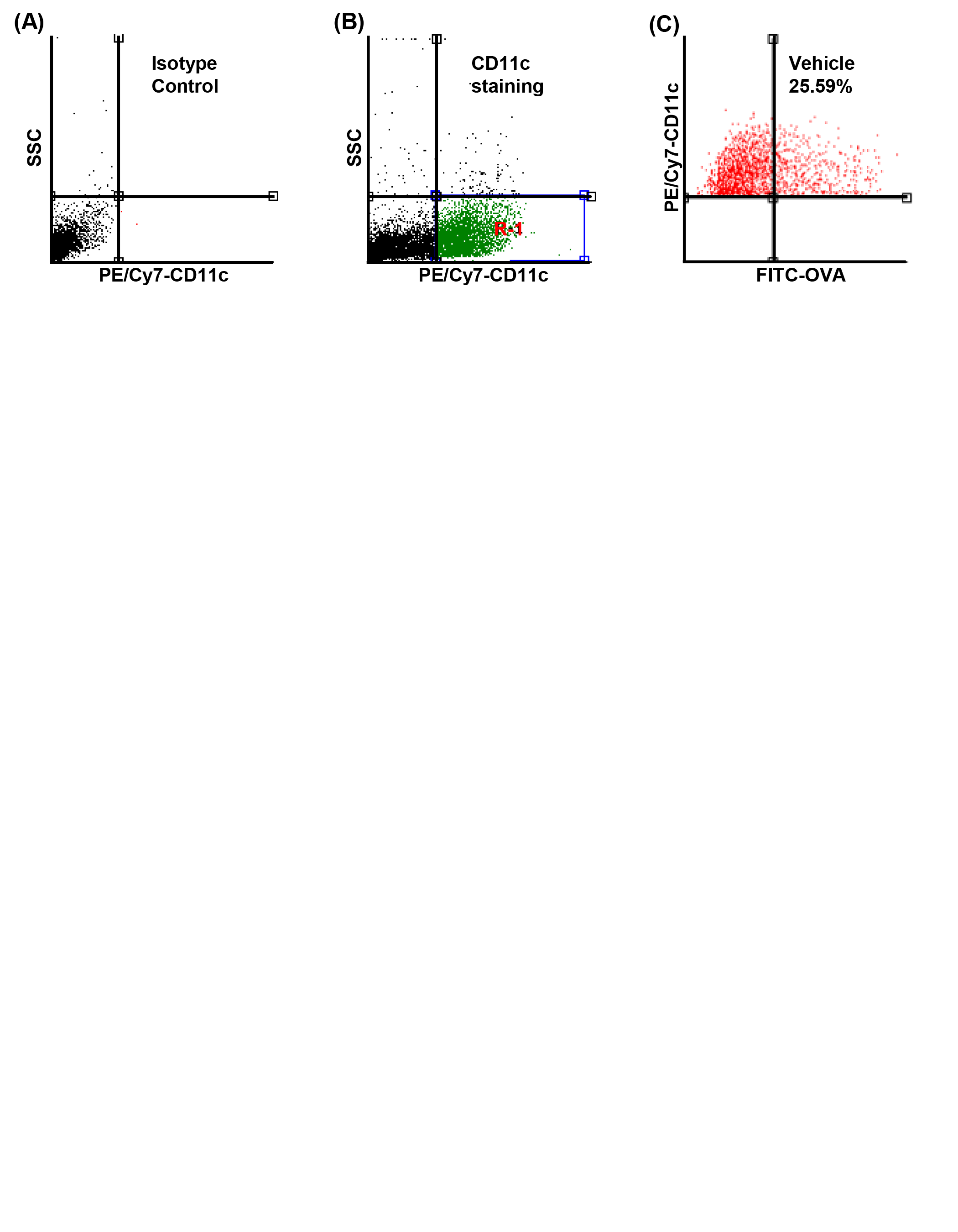

Supplement: Figure S1 — Gating strategy used for multicolor flow cytometry analysis. WT bone marrow-derived cells were incubated with FITC-OVA for 5 h, stained either with PE/Cy7-labeled CD11c (B) or respective IgG control (A) monoclonal antibodies, fixed in 2% paraformaldehyde, and processed for flow cytometry analysis. The cells were first gated on size and then doublets excluded using forward scatter area and forward scatter height. Cells were gated on CD11c+ population to identify dendritic cells (R1; green). Ovalbumin internalization was determined by measuring FITC fluorescence in R1 population (green) (C). [file image_1.TIF]

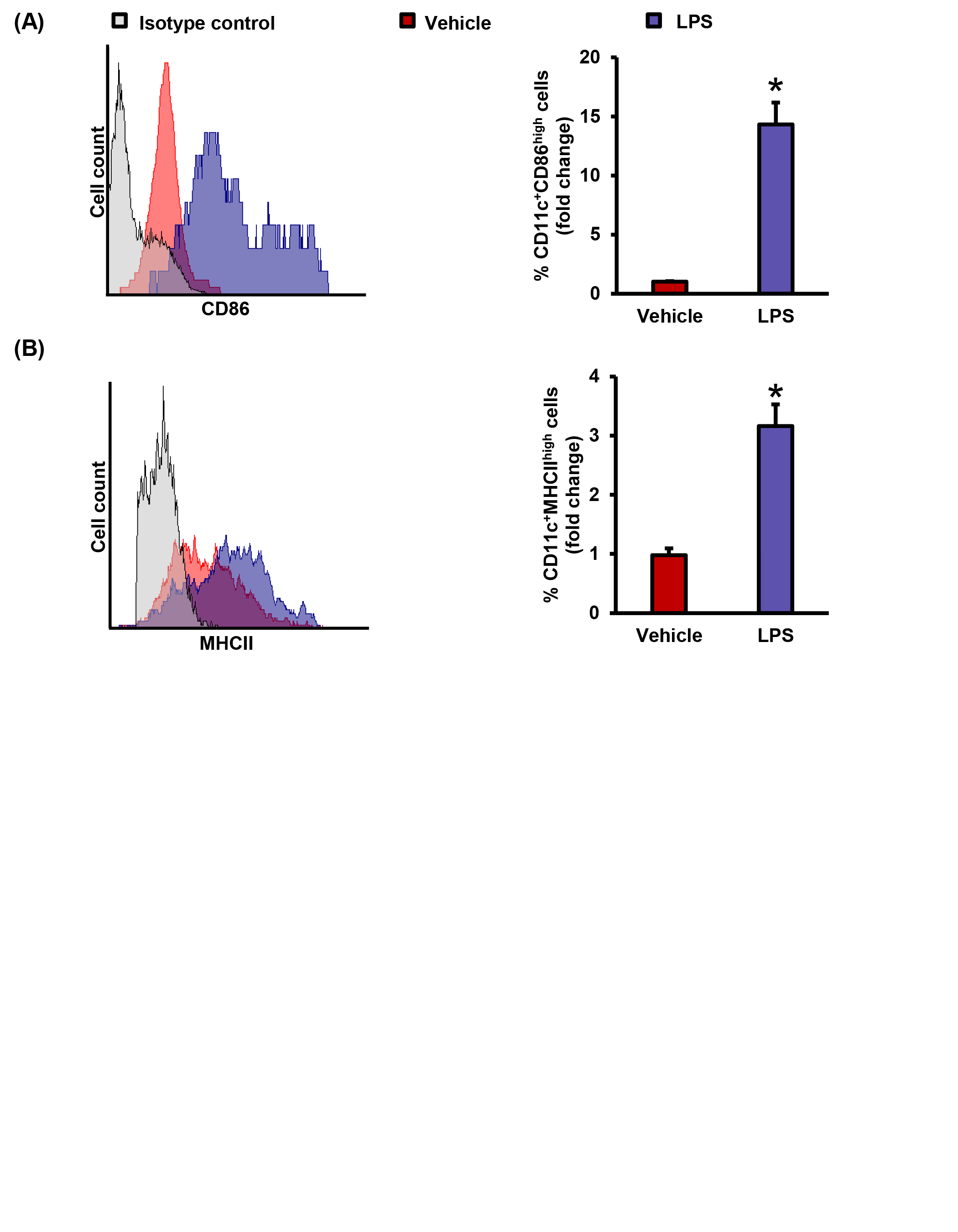

Supplement: Figure S2 — Confirmation of immature phenotype of BMDCs. CD11c+ cells were incubated with vehicle or lipopolysaccharide (LPS) (500 ng/mL) for 24 h, fixed, and incubated with FITC-MHCII (clone M5/114.15.2), APC-CD86 (clone GL1) or respective IgG control monoclonal antibodies. CD86 (A) and MHC II (B) expression in CD11c+ dendritic cells were determined using flow cytometry. *p < 0.05 vs. vehicle. [file image_2.TIF]

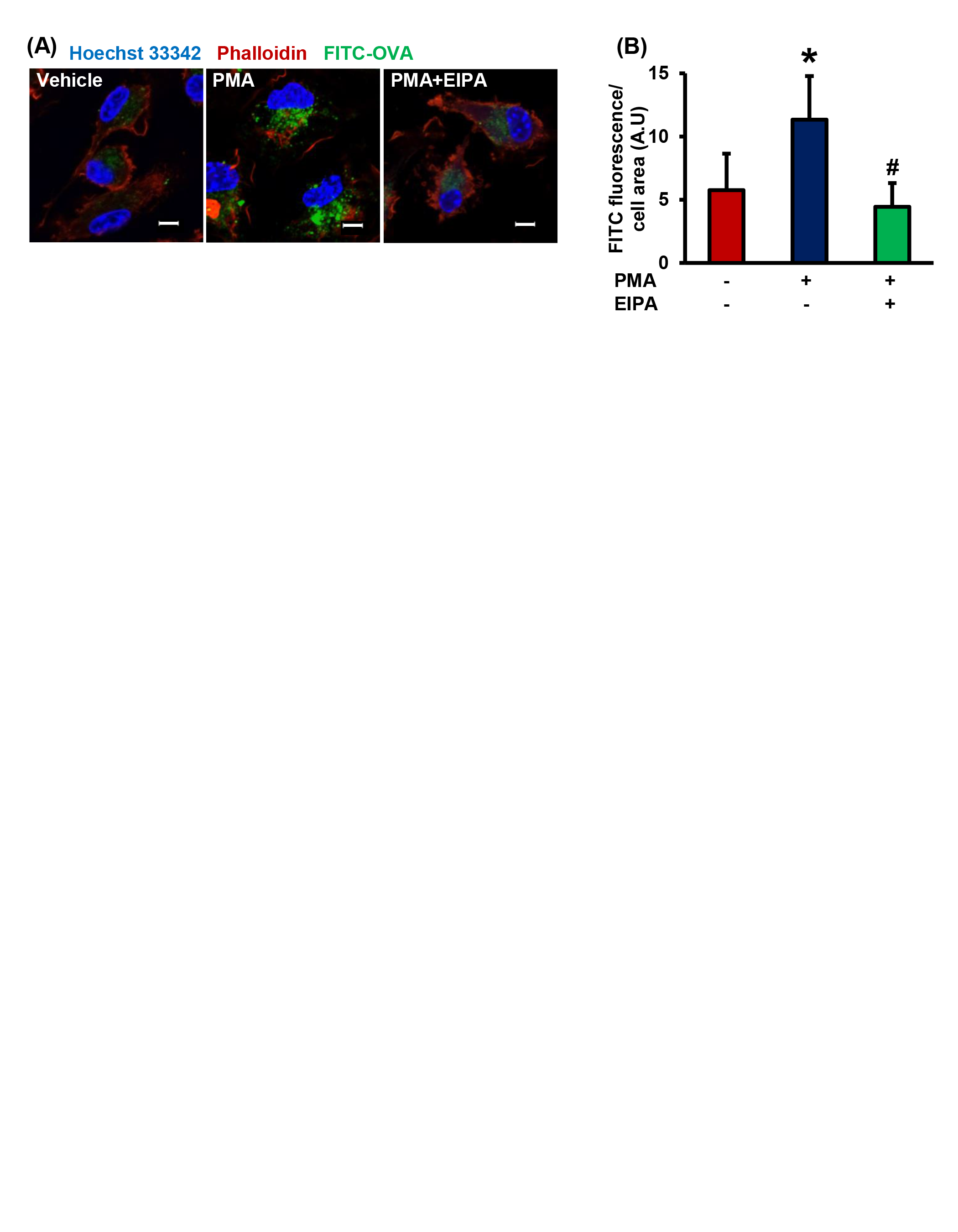

Supplement: Figure S3 — EIPA inhibits FITC-OVA uptake in bone marrow-derived immature dendritic cells (BMiDCs). (A) Wild-type (WT) CD11c+ BMiDCs were treated with vehicle or phorbol 12-myristate 13-acetate (PMA, 1 µM, 5 h), or pretreated with EIPA (25 µM, 30 min.) followed by PMA treatment in the presence of FITC-OVA. FITC-OVA internalization (green) was analyzed by confocal microscopy. Scale bar: 5 µm. (B) Bar diagram shows quantified FITC fluorescence intensity normalized to cell area (n = 3). At least five images per treatment were analyzed for each independent experiment using Image J. *p < 0.05 vs. vehicle, #p < 0.05 vs. PMA. [file image_3.TIF]

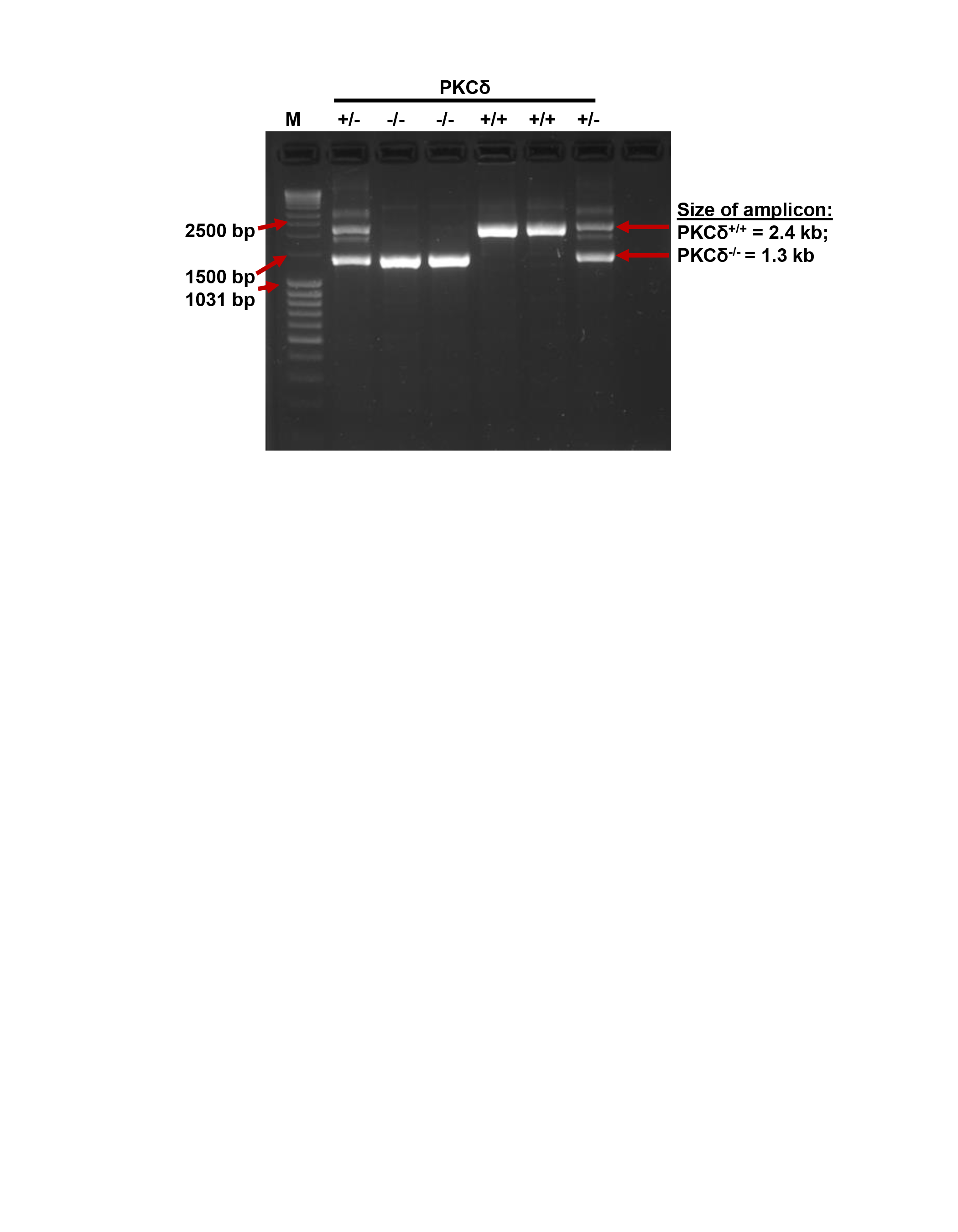

Supplement: Figure S4 — Representative Agarose gel electrophoresis image showing mice genotyping for PKCδ expression. [file image_4.TIF]

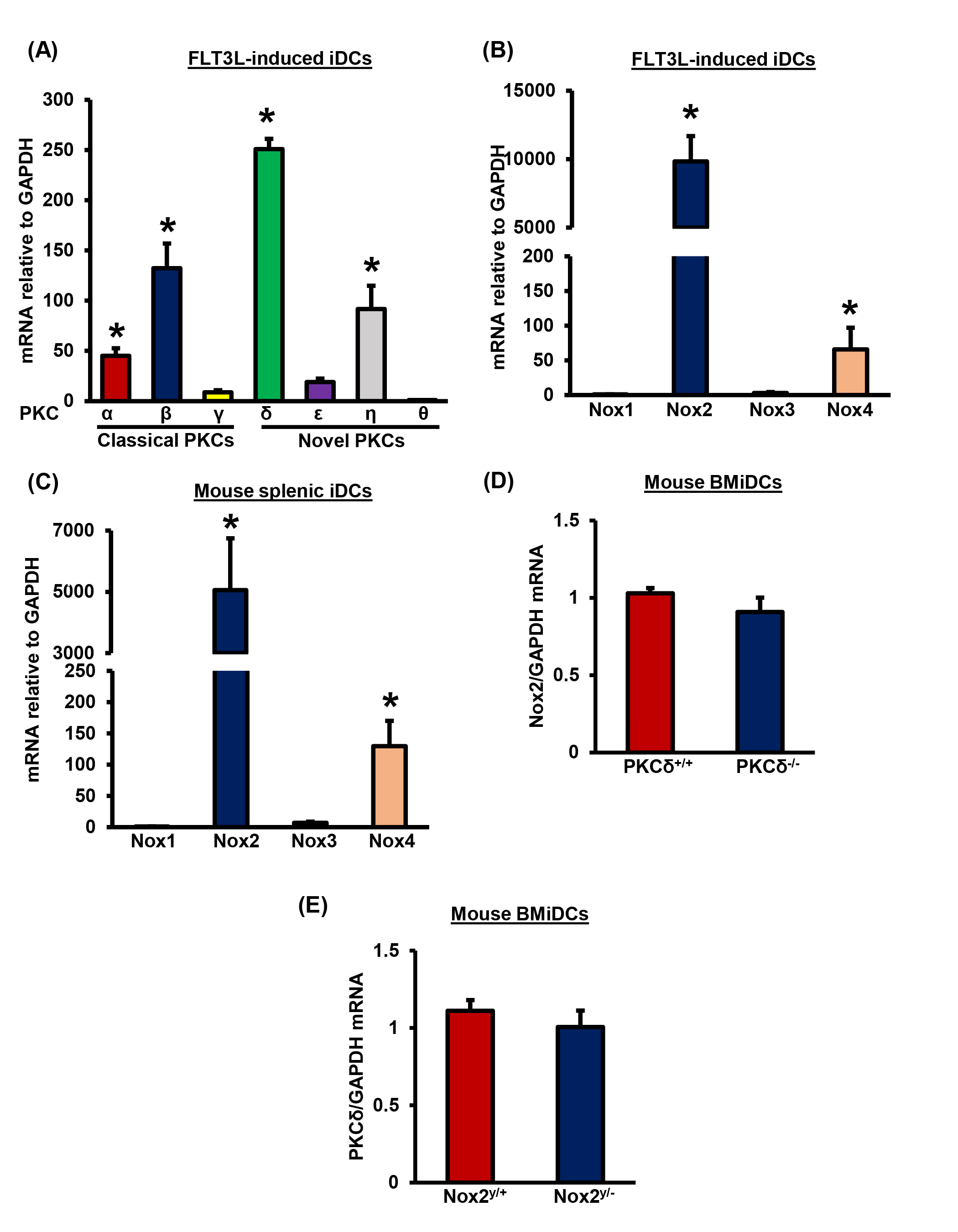

Supplement: Figure S5 — PKCδ and Nox2 is the dominant PKC and Nox isoform in FLT3L-induced immature DCs (iDCs). (A) WT FLT3L-differentiated CD11c+ iDCs were subjected to qRT-PCR for determination of various PKCs levels. GAPDH was used as an internal control. Data are representative of three independent experiments performed in triplicate. Bar graph represents mRNA levels in comparison to PKCθ. *p < 0.05 vs. PKCθ. (B) Transcript levels of Nox1, Nox2, Nox3, and Nox4 in WT FLT3L-differentiated CD11c+ iDCs. Bar graph represents mRNA levels in comparison to Nox1 (gene with lowest expression). *p < 0.05 vs. Nox1. (C) Nox1, Nox2, Nox3, and Nox4 mRNA expression in WT CD11c+ splenic iDCs. *p < 0.05 vs. Nox1. (D) PKCδ expression in Nox2y/+ and Nox2y/− BMiDCs (n = 3). (E) Nox2 expression in PKCδ+/+ and PKCδ−/− BMiDCs (n = 3). Data represent the mean ± SEM (A–E). [file image_5.TIF]

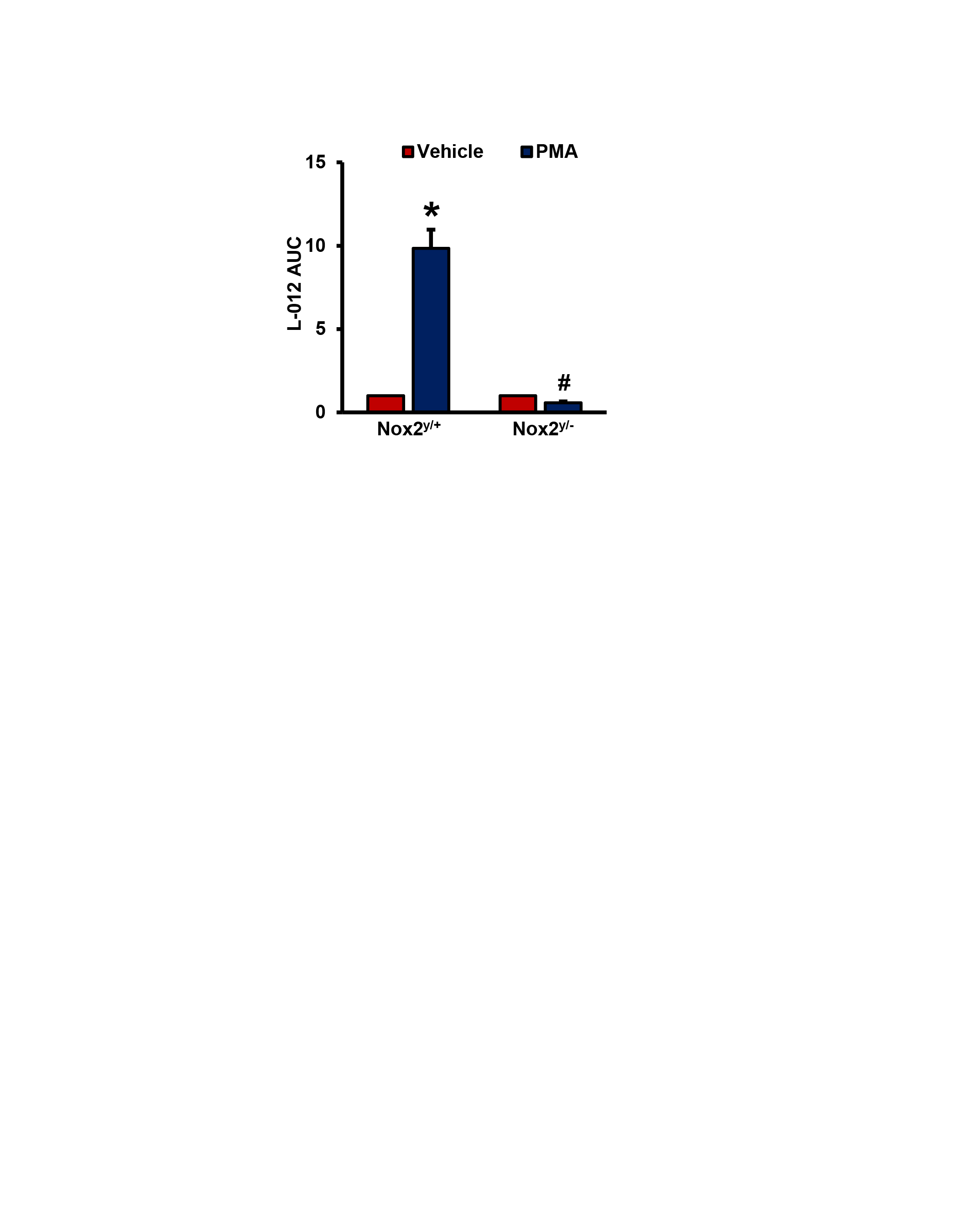

Supplement: Figure S6 — Nox2 activation stimulates reactive oxygen species production in iDCs. Nox2y/+ (wild type) and Nox2y/− BMiDCs were treated with vehicle or PMA (1 µM) and O2 •− production was monitored using L-012 chemiluminescence. The diagram represents the mean fold change of L-012 chemiluminescence area under the curve obtained in three independent experiments. *p < 0.05 vs. vehicle, #p < 0.05 vs. Nox2y/+ PMA. [file image_6.TIF]

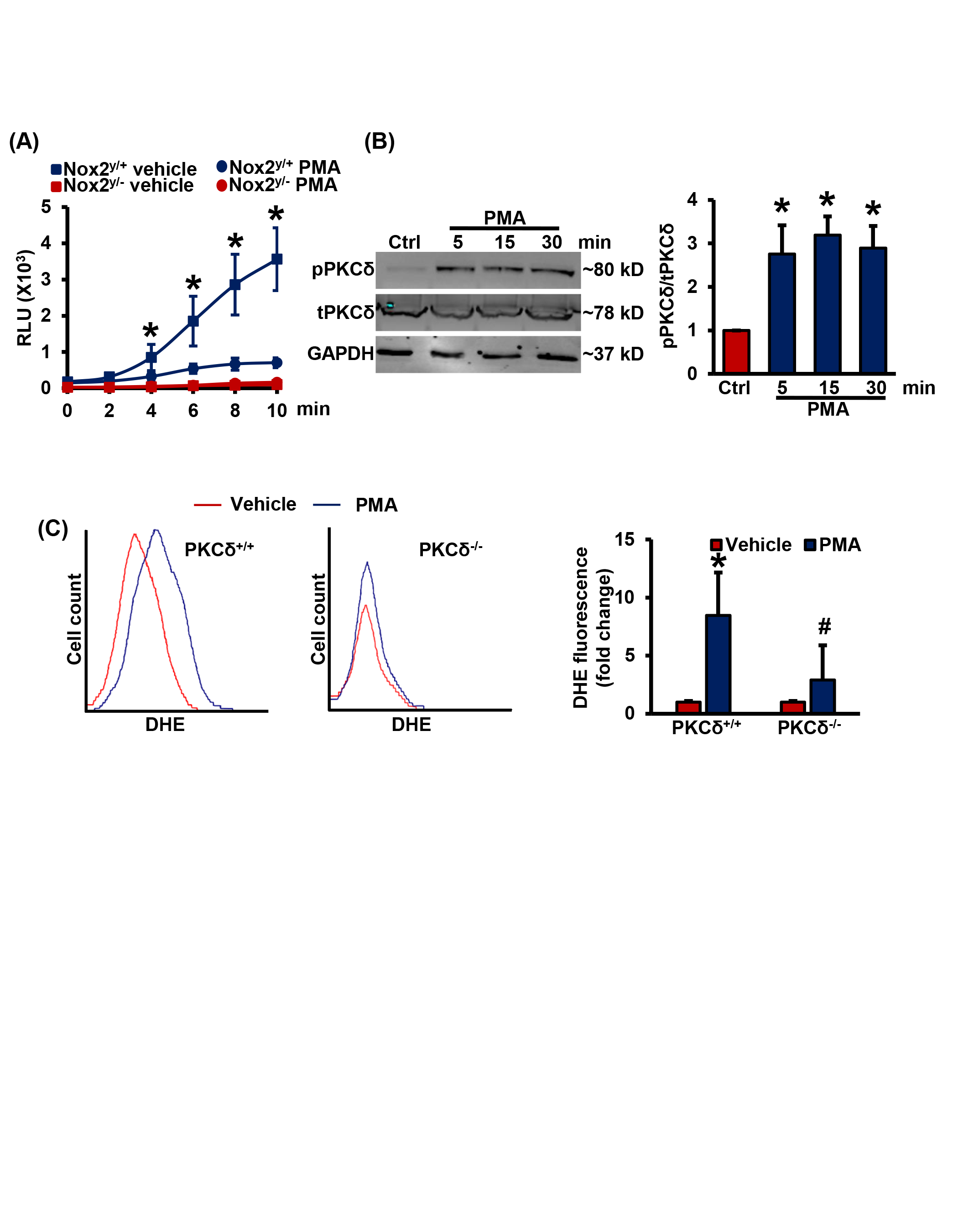

Supplement: Figure S7 — Stimulation of Nox2-derived O2 •− generation in PMA-treated immature DCs precedes or occurs simultaneously with PKCδ activation. (A) O2 •− generation was measured in vehicle- and PMA-treated Nox2y/+ (wild type) and Nox2y/− BMiDCs using L-012 chemiluminescence (n = 3). (B) WT BMiDCs were treated with vehicle (30 min) or incubated with PMA (1 µM) for 5, 15, and 30 min. Cells were lysed and subjected to Western blot analysis for phospho-PKCδ (tyr311), total PKCδ, and GAPDH protein expression. Representative Western blot images are shown. The bar graph represents the ratio of phospho- to total PKCδ from three to six independent experiments. (C) Dihydroethidium (DHE) fluorescence was quantified in vehicle- and PMA (1 µM, 30 min.)-treated PKCδ+/+ and PKCδ−/− BMiDCs using fluorescence-activated cell sorting analysis. Representative histograms showing DHE fluorescence are presented (left and middle panels). Bar diagram indicates fold change in mean fluorescence intensity (n = 3). *p < 0.05 vs. vehicle, #p < 0.05 vs. PKCδ+/+ PMA. [file image_7.TIF]

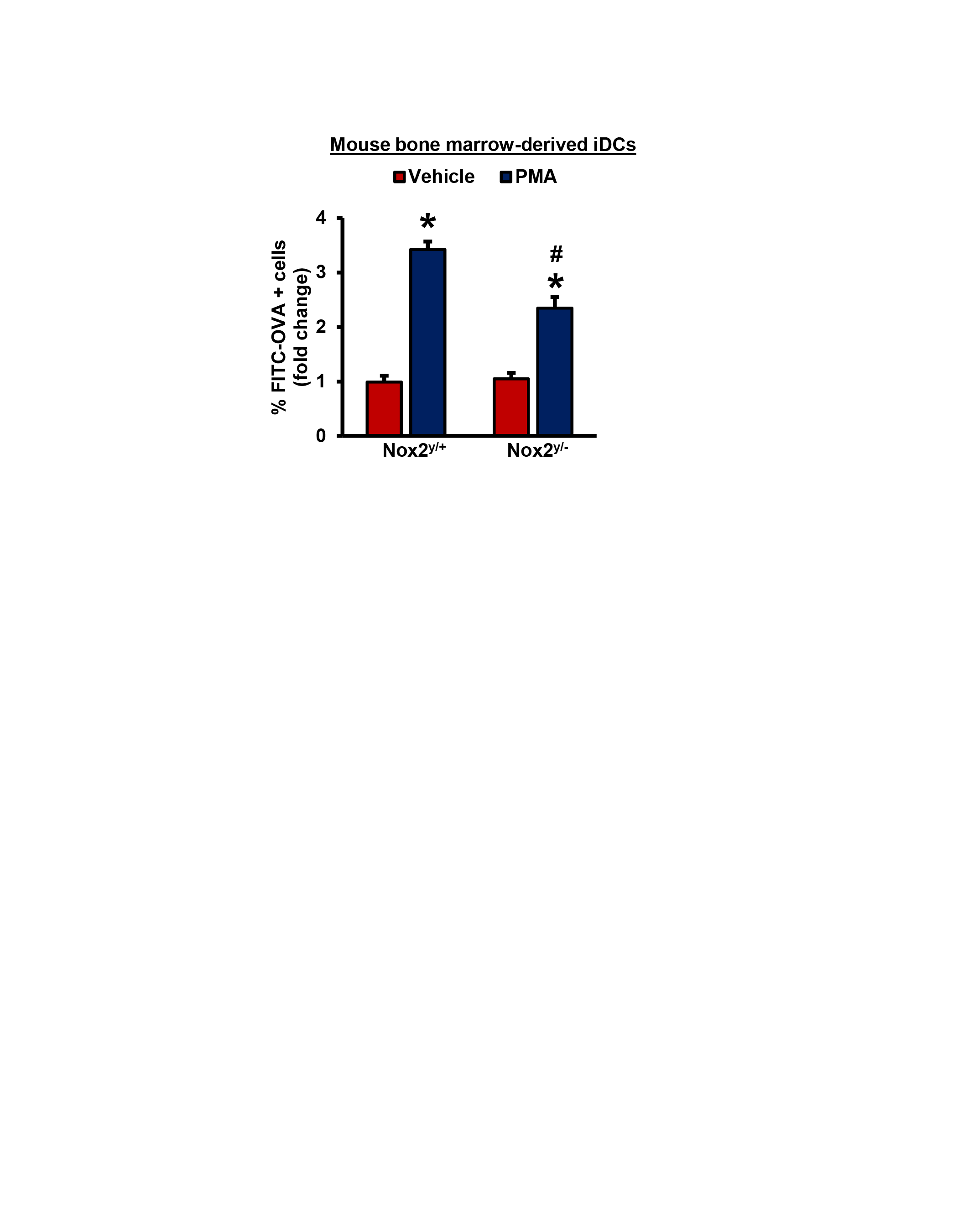

Supplement: Figure S8 — Loss of Nox2 in BMiDCS attenuates PMA-stimulated macropinocytosis of ovalbumin (OVA). Nox2y/+ and Nox2y/− BMiDCs were incubated with vehicle or stimulated with PMA (1 µM, 5 h) in the presence of FITC-OVA. FITC fluorescence was analyzed in CD11c+ cells using fluorescence-activated cell sorting (FACS) (n = 3). *p < 0.05 vs. vehicle, #p < 0.05 vs. Nox2y/+ PMA. [file image_8.TIF]

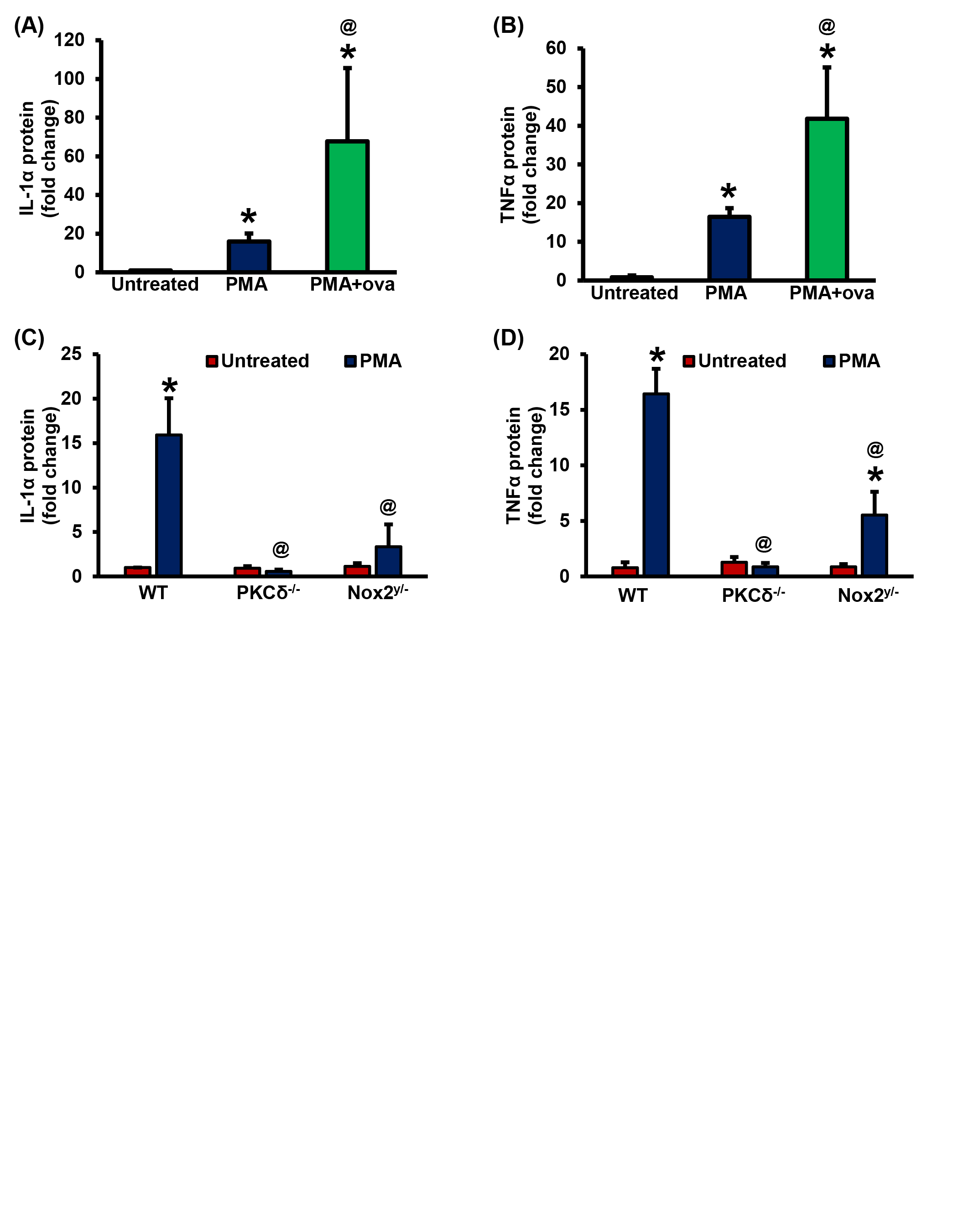

Supplement: Figure S9 — “PMA + OVA” treatment induces a more pronounced secretion of DC-derived T-cell regulatory cytokines compared to PMA treatment alone. (A–B) WT BMiDCs left untreated or treated with PMA or PMA + OVA for 24 h. Cell culture supernatants were collected and levels of secreted cytokines determined using the LEGENDplex™ bead-based immunoassay. *p < 0.05 vs. vehicle, and @p < 0.05 vs. PMA. (C–D) WT, PKCδ−/−, and Nox2y/− BMiDCs were left untreated or treated with PMA for 24 h. Cell culture supernatants used for cytokine estimation as described in (A–B). Data are representative of three independent experiments and values are expressed as mean ± SD. *p < 0.05 vs. vehicle and @p < 0.05 vs. WT PMA. [file image_9.TIF]
