# Supplementary material for: PKCδ-Mediated Nox2 Activation Promotes Fluid-Phase Pinocytosis of Antigens by Immature Dendritic Cells
Source: Front Immunol. 2018 Mar 26;9:537. doi: 10.3389/fimmu.2018.00537 (PMC5879126; doi:10.3389/fimmu.2018.00537)
Supplement: Table S1 — List of primers used for mRNA quantitation using real-time-PCR. [file table_1.PDF]

Supplementary Table 1: List of primers used for mRNA quantitation using real-time-PCR.

Gene name	Primer sequences
**PKCα**	F 5’-CCCATTCCAGAAGGAGATGA-3’<br>R 5’-TTCCTGTCAGCAAGCATCAC-3’
**PKCβ**	F 5’-TCCCTGATCCCAAAAGTGAG-3’<br>R 5’-AACTTGAACCAGCCATCCAC-3’
**PKCγ**	F 5’-ACCAGGGCATCATCTACAGG-3’<br>R 5’-CTTCCTCATCTTCCCCATCA-3’
**PKCδ**	F 5’-CAGACCAAGGACCACCTGTT-3’<br>R 5’-GCATAAAACGTAGCCCGGTA-3’
**PKCε**	F 5’-GAGGACTGGATTGACCTGGA-3’<br>R 5’-ATCTCTGCAGTGGGAGCAGT-3’
**PKCη**	F 5’-CATCCCACACAAGTTCAACG-3’<br>R 5’-ATATTTCCGGGTTGGAGACC-3’
**PKCθ**	F 5’-ATGGACAACCCCTTCTACCC-3’<br>R 5’-GCGGATGTCTCCTCTCACTC-3’
**Nox1**	F 5’-TGGCTAAATCCCATCCAGTC-3’<br>R 5’-CCCAAGCTCTCCTCTGTTTG-3’
**Nox2**	F 5’-TGAATGCCAGAGTCGGGATTT -3’<br>R 5’-CGAGTCACGGCCACATACA -3’
**Nox3**	F 5’-CTCGTTGCCTACGGGATAGC-3’<br>R 5’-CCTTCAGCATCCTTGGCCT-3’
**Nox4**	F 5’-ACTTTTCATTGGGCGTCCTC-3’<br>R 5’-AGAACTGGGTCCACAGCAGA-3’
**IL-6**	F 5’-CACAAGTCGGAGGCTTAAT-3’<br>R 5’-GTGCATCATCGTTGTTCATAC-3’
**IL-1α**	F 5’-CCCGTCCTTAAAGCTGTCTG-3’<br>R 5-AATTGGAATCCAGGGGAAAC-3’
**TNF-α**	F 5’-CTGGGACAGTGACCTGGACT-3’<br>R 5’-GCACCTCAGGGAAGAGTCTG-3’
